# Optoregulated mRNA Delivery Controls Pleiotropic Immune Signaling for Tumor‐Targeted Therapy

**DOI:** 10.1002/anie.202513610

**Published:** 2025-09-02

**Authors:** Pengwen Chen, Guanghao Hu, Yuki Nakashima, Zhining Xu, Takayoshi Watanabe, Soichiro Kondo, Ervin Kovács, Horacio Cabral

**Affiliations:** ^1^ Department of Bioengineering Graduate School of Engineering The University of Tokyo 7‐3‐1 Hongo Bunkyo‐ku Tokyo 113‐0033 Japan; ^2^ Institute of Materials and Environmental Chemistry HUN‐REN Research Centre for Natural Sciences 1117, Magyar Tudósok Körútja 2 Budapest Hungary; ^3^ Hevesy György PhD School of Chemistry Eötvös Loránd University 1053, Egyetem tér 1–3 Budapest Hungary

**Keywords:** Endosomal escape, Immunotherapy, Interleukin‐2, Messenger RNA, Photosensitizer

## Abstract

Spatiotemporal control of protein expression remains a critical challenge in messenger RNA (mRNA) therapeutics, particularly for tumor‐targeted therapy. Here, we introduce a Light‐Induced Transfection System (LITS) leveraging photosensitizing polymers to deliver mRNA systemically and activate its translation via light‐triggered endosomal escape. This system enables localized protein expression in any irradiated tissue, allowing for spatial control of therapeutic effects. Using interleukin‐2 (IL‐2) as a model, we demonstrate that LITS can trigger proinflammatory cytokine levels in irradiated tumors, while inducing tolerogenic IL‐2 levels in nonirradiated healthy tissues. This modulation of IL‐2′s pleiotropic effects provides both potent antitumor activity and reduced toxicity. Furthermore, by optimizing light exposure, LITS synergizes IL‐2 efficacy by driving immunogenic cell death to eradicate lung metastasis in a breast cancer model. Our findings establish LITS as a programmable mRNA delivery platform for on‐demand targeted and safe therapies.

## Graphical abstract

A light‐induced transfection system (LITS) was designed to precisely control the delivery of mRNA, achieving spatiotemporal de‐coupling of the pleotropic inflammatory and tolerogenic signals of interleukin‐2 to conduct safe eradication of tumors.

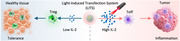

## Introduction

Messenger RNA (mRNA) technology has proven successful in applications such as COVID‐19 and cancer vaccines^[^ ^1^, ^2^, ^3^ ^]^ showing its potential to revolutionize medicine. However, its broader adoption is hampered by the limitations of current delivery methods in achieving precise control of protein expression across diverse tissues and timeframes.^[^ ^4^, ^5^, ^6^, ^7^ ^]^ Indeed, despite significant efforts to improve the biodistribution and cellular interaction, systemic mRNA delivery still struggles with targeting and regulating protein levels beyond the organs of the reticuloendothelial system (RES).^[^ ^8^, ^9^, ^10^, ^11^, ^12^, ^13^, ^14^, ^15^, ^16^, ^17^ ^]^ These challenges highlight the need for delivery systems capable of precise spatiotemporal control of protein expression.

Light‐mediated delivery is a powerful strategy that enables targeted gene therapy through pinpoint irradiation.^[^ ^18^, ^19^, ^20^, ^21^ ^]^ This approach holds promise for enhancing protein expression by controlling intracellular trafficking, such as endosomal escape and cytosolic access.^[^ ^22^, ^23^ ^]^ For instance, incorporating photothermal lipids into LNPs increased mRNA transfection in the liver and spleen under light stimulation.^[^ ^19^ ^]^ Yet, achieving light‐controlled mRNA delivery to tissues beyond the RES, including tumors, is still challenging and rarely reported. Overcoming this obstacle requires the development of innovative delivery systems capable of effective distribution to non‐RES tissues. These systems must also feature enhanced photosensitivity to enable precise and efficient mRNA release with minimal light exposure for maintaining mRNA integrity, and improving safety and therapeutic outcomes.

**Figure 1:**
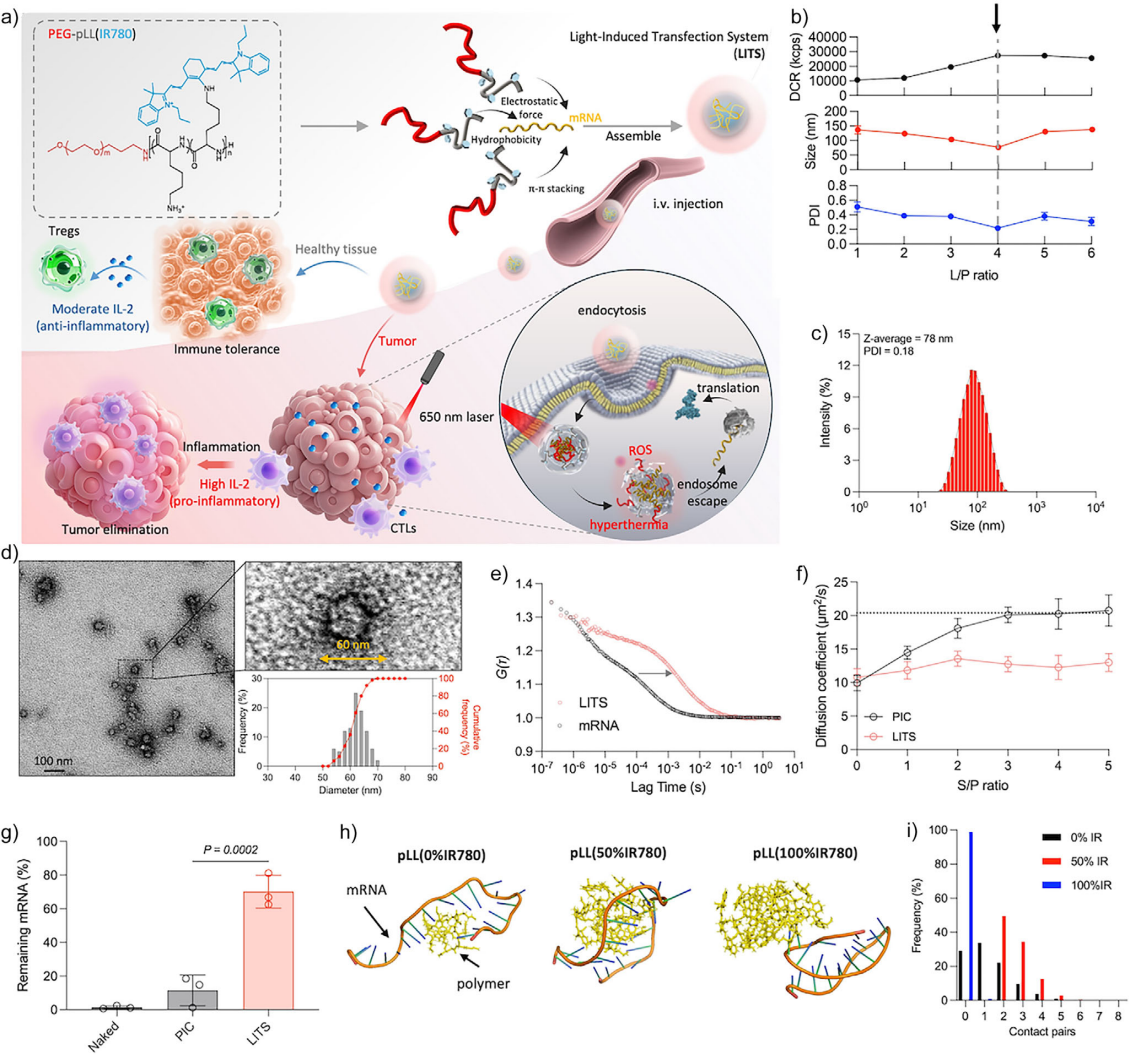
Photosensitizing polymers stably encapsulate mRNA in core‐shell complexes. a) LITS is composed of photosensitizing polymers complexed with mRNA, forming compact sub‐100 nm nanoparticles. Systemically injected LITS delivers mRNA to major organs and tumors. After cellular uptake, light irradiation activates the polymer, generating localized hyperthermia, and reactive oxygen species (ROS), which facilitate endosomal escape of mRNA and enhance protein expression. This optoregulated mRNA transfection enables precise control of pleiotropic cytokine signaling pathways, such as IL‐2, promoting strong proinflammatory responses in irradiated tumors and anti‐inflammatory effects in nonirradiated tissues. b) DLS measurement results of LITS formed with different *L/P* ratio. The light scattering intensity (DCR, upper panel), size (middle panel), and PDI (lower panel) were recorded to indicate the formation of particles. Data are plotted as the mean ± S.D., *n *= 3 independent measurements. c) Representative size distribution of LITS at *L/P* = 4. d) Representative TEM image of LITS (*L/P* = 4). Size of the stained spherical structure was summarized in the histogram. *n *= 100 particles analyzed. e) Representative ACF curves of free mRNA and LITS (*L/P* = 4) measured by FCS. f) FCS measurement results of LITS or PIC incubated with dextran sulphate. Data are plotted as the mean ± S.D., *n *= 3 independent measurements. The dotted line (*y* = 20.4 µm^2^ s^−1^) indicates the diffusion coefficient of free Luc‐mRNA. g) The remaining amount of mRNA after incubation with FBS measured by RT‐PCR. Data are plotted as the mean ± S.D., *n *= 3 independent samples. The results were compared via one‐way ANOVA. h) Molecular dynamic simulation results showing the interactions between the polymers with mRNA. i) The contact pairs between the polymers and mRNA during the simulation time duration.

Herein, we present an optoregulated strategy for selective mRNA delivery to targeted tissues using a Light‐Induced Transfection System (LITS) (Figure 1a). This system employs photosensitizing polymers specifically designed for engaging with mRNA molecules via multiple noncovalent interactions. These include π–π stacking mediated by the hydrophobic photosensitizer IR780, and ion complexation with IR780 and lysine residues in the poly(L‐lysine) segment of poly(ethylene glycol)‐poly(L‐lysine) (PEG‐pLL) block copolymers. These interactions form densely PEGylated nanoparticles with sub‐100 nm diameter that encapsulate mRNA within a stable core. This design allows efficient delivery of mRNA to major organs and tumor tissues after systemic administration. Upon endocytosis, LITS responds to the acidic endosomal environment by releasing mRNA and activating the photosensitizing effects of the polymer. Subsequent light irradiation excites the polymer to generate hyperthermia and reactive oxygen species (ROS) that disrupt endosomal membranes, facilitating endosomal escape and enabling precise control over protein expression by adjusting irradiation conditions.

To demonstrate LITS potential, we focused on optoregulating the pleiotropic signals of interleukin‐2 (IL‐2). Specifically, high IL‐2 levels promote proinflammatory signaling to enhance T cell‐mediated antitumor immunity, whereas low doses selectively expand regulatory T cells (Tregs) to support anti‐inflammatory responses.^[^ ^24^, ^25^ ^]^ When delivered via LITS, IL‐2 mRNA effectively transfected tumor cells after irradiation, yielding elevated intratumoral IL‐2 expression, and only marginally increased IL‐2 levels in nonirradiated tissues. This precise control spatially decoupled the pleotropic effects of IL‐2, simultaneously enhancing the antitumor efficacy while maintaining the immune tolerance in healthy tissues. Moreover, prolonged irradiation induced immunogenic cell death (ICD) in tumors, which synergized with the tumor‐targeted IL‐2 expression to amplify the therapeutic effects. These findings underscore the potential of LITS for antitumoral mRNA therapy using optoregulation to precisely modulate immune response.

## Results and Discussion

### Photosensitizing Polymers Stably Encapsulate mRNA in Core‐Shell Complexes

IR780 was conjugated to the lysine moieties of PEG‐pLL to prepare the photosensitizing polymers (Scheme S1). The pLL block contains 80 lysine units, with IR780 modifying 38 of these units. (Figures S1–S3). Size exclusion chromatography (SEC) confirmed the monodisperse molecular weight distribution of the polymers (Figure S4).

The photosensitizing polymers exhibited pH‐dependent solubility. Dynamic light scattering (DLS) analysis revealed that the polymers associated into particles of around 44 nm in neutral buffer (pH = 7.4), while in acidic buffer (pH = 4.5) the polymers remained dissociated with a size around 10 nm and low light scattering intensity measured as derived count rate (DCR) (Figure S5). This pH‐dependent solubility was attributed to the protonation of the secondary amines generated after IR780 conjugation, which increases hydrophilicity and enhances electrostatic repulsion between the polymers at acidic pH. Consequently, the polymer stock solution was prepared in acidic buffer (pH 4.5) to prevent premature association before assembling the LITS.

mRNA‐loaded LITS formation was screened by mixing polymer and mRNA solutions in buffer (10 mM HEPES, pH 7.4) with varying ratios of lysine units in the polymer to phosphate groups in mRNA, i.e., *L/P* ratios. Gel electrophoresis demonstrated that by increasing the polymer concentration in the mixture, remaining free mRNA was diminished (Figure S6A), indicating the loading of mRNA into the LITS. DLS measurements also confirmed the formation of nanoparticles for *L/P* ratios higher than one (Figure 1b). At *L/P* ratios = 4 and above, the DCR reached a plateau, which indicates that no additional nanoparticles were formed, suggesting the complete assembly of LITS. Also, *L/P* ratio = 4 resulted to a complete encapsulation of mRNA (>95%) (Figure S6B). Therefore, *L/P* ratio = 4 was selected to formulate the LITS for the following studies. This LITS was monodisperse (PDI < 0.2) with an average hydrodynamic size of around 80 nm (Figure 1c). Transmission electron microscopy (TEM) images showed nanoparticles with an average diameter of around 60 nm (Figure 1d). Considering the PEG shell has low electron density, which makes it hard to be visualized by TEM, these particles are assumed to represent the cores of the LITS. Also, the difference of the size measured by DLS and that obtained by TEM suggests that the LITS has a 10 nm thick PEG shell, confirming the core‐shell structure. The dense PEG shell shielded the charge of the core, resulting in a nearly neutral *ζ*‐potential (Figure S7). Fluorescence correlation spectroscopy (FCS) further confirmed mRNA encapsulation within the LITS. Compared to free mRNA, mRNA encapsulated in LITS showed a lagged autocorrelation function (ACF) curve (Figure 1e), indicating delayed diffusion due to its relatively larger size (Table S1). Moreover, LITS and free mRNA showed similar count per molecule values, which indicates that there is one mRNA molecule loaded per LITS particle.

The stability of LITS was evaluated in various conditions and compared to mRNA/PEG‐pLL polyion complex (PIC) to understand the effects of IR780 conjugation. The PIC was prepared at the same *L/P* ratio of LITS (*L/P* = 4), forming complexes of around 120 nm. The characterization of PIC is summarized in Figure S8. Adding an electrolyte, i.e., NaCl, into the buffer destabilized the PIC, as its formation is mainly driven by electrostatic interaction between the amines in the lysine moieties and the phosphate groups in mRNA (Figure S9). In contrast, LITS were stable even in 500 mM NaCl, maintaining the monodisperse size. Moreover, PICs are prone to dissociation through polyion exchange when exposed to anionic macromolecules in physiological environment, such as glycosaminoglycans on cell surfaces.^[^ ^26^, ^27^ ^]^ Such premature release leads to the degradation of mRNA in extracellular space. Thus, we investigated the dissociation of PICs and LITS using dextran sulphate as a model polyanion. Samples were incubated at varying concentrations of dextran sulphate to achieve different ratios between the sulphate in dextran sulphate and the phosphate in mRNA (*S/P*). FCS analysis revealed that PICs started to dissociate at *S/P* = 1, as indicated by an increase in the diffusion coefficient (Figure 1f). At *S/P* = 3, PIC completely dissociated to release the mRNA. In contrast, LITS remained stable even at *S/P* = 5, confirming its enhanced stability in the presence of polyanions. The stability of LITS was also evaluated by FCS in buffer containing 10% FBS to simulate the physiological condition. LITS displayed negligible changes in the diffusion coefficient, indicating they can maintain the structure in the buffer (Figure S10). In contrast, PICs showed a gradual increase in the diffusion coefficient, inferring their instability. The ability of the formulations to protect mRNA from nucleases in physiological environment was then evaluated by reverse transcription PCR (RT‐PCR). After 30 min incubation in 50% fetal bovine serum (FBS), both unencapsulated mRNA and the mRNA in PIC were degraded (Figure 1g), whereas LITS protected over 70% of the mRNA. These results suggest that LITS stability could be suitable for delivering mRNA under physiological conditions.

To elucidate the role of IR780 in the enhanced stability of LITS, we investigated the interactions between the polymer and mRNA by molecular dynamics simulations using three pLL derivatives with varying degrees of IR780 conjugation, i.e., 0%, 50%, and 100% of lysine residues modified. These derivatives were designated as pLL(0%IR780), pLL(50%IR780), and pLL(100%IR780), respectively. pLL(50%IR780), which represents the structure of the polymer used in LITS, exhibited more compact binding and stable interactions with mRNA than pLL(0%IR780) (Figure 1h,i and Videos S1–S3). This was evidenced by a reduced radius of gyration and stable root mean square deviation (RMSD) of the molecules (Figure S11). Increasing the IR780 conjugation to 100% lead to an extremely coiled polymer structure that is unable to interact with mRNA (Figure 1h,i and Videos S1–S3). These observations suggest that the partial IR780 substitution on the side chain of PEG‐pLL can enhance the polymer‐mRNA interactions, thereby improving complex stability.

### LITS Allows pH‐Dependent Photosensitizing Effects

**Figure 2:**
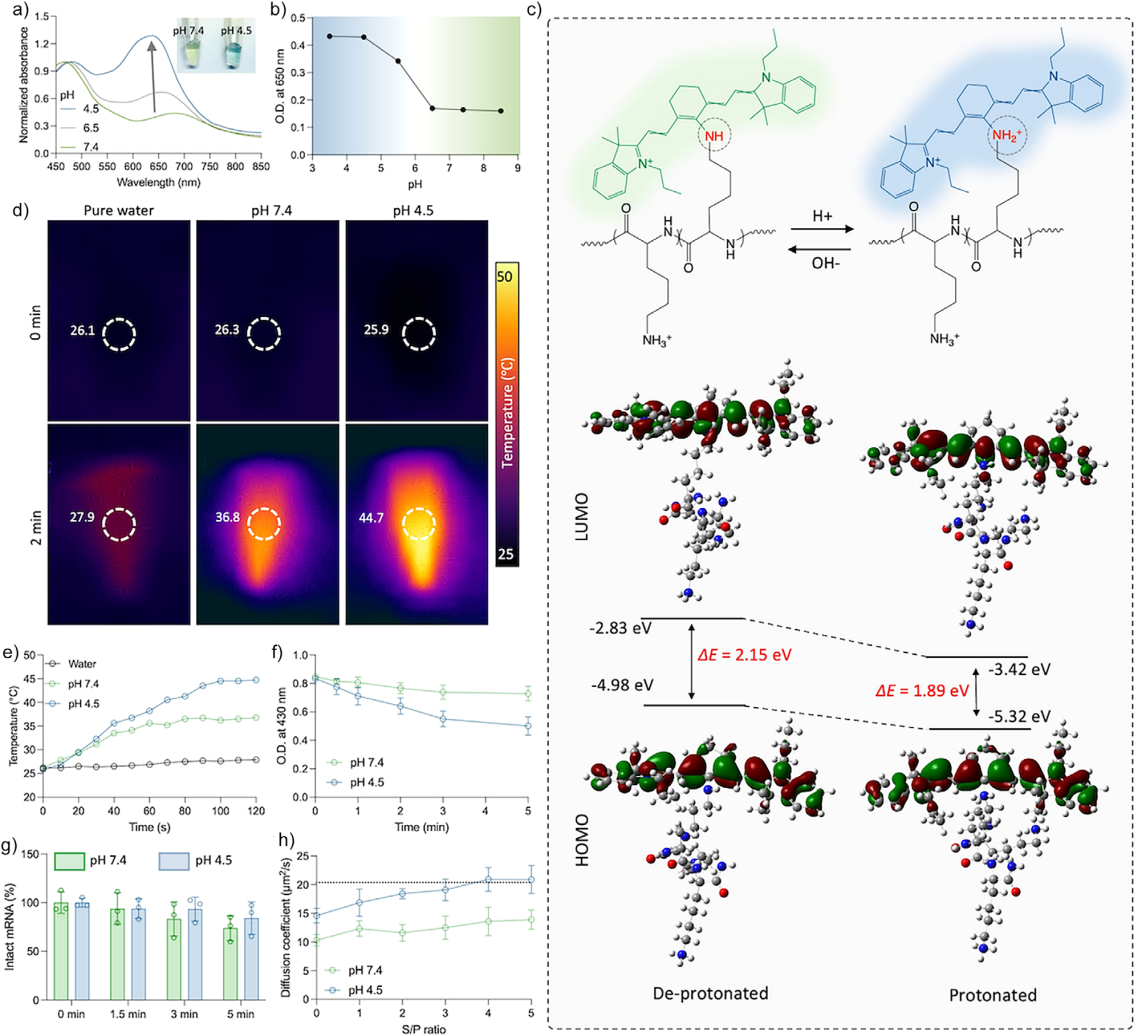
LITS allows pH‐dependent photosensitizing effects. a) Absorbance spectra of PEG‐pLL(IR780) dissolved in buffers with different pH. Inserted photos show the polymer solution at pH 4.5 and 7.4. b) Absorbance at 650 nm of the polymer dissolved in buffers with different pH. Data are plotted as the mean ± S.D., *n *= 3 independent measurements. c) DFT computation results showing electron distribution and energy level of the molecular orbitals. d) Representative thermal images of LITS in buffers with different pH after 650 nm irradiation. e) Temperature of LITS solution during 650 nm laser irradiation. Pure water was used as the blank comparison. f) ROS generation in LITS solution during 650 nm laser irradiation determined by 1,3‐diphenylisobenzofuran (DPBF) probe test. The absorbance at 430 nm of the sample was measured to indicate the oxidation of DPBF. Data are plotted as the mean ± S.D., *n *= 3 independent measurements. g) Integrity of mRNA loaded in LITS after irradiation with 650 nm laser for different time. Data are plotted as the mean ± S.D., *n *= 3 independent samples. h) FCS measurement results of LITS incubated with dextran sulphate under different pH. Data are plotted as the mean ± S.D., *n *= 3 independent measurements. The dotted line (*y* = 20.4 µm^2^ s^−1^) indicates the diffusion coefficient of free Luc‐mRNA.

Besides the solubility of the polymers, the photosensitizing properties also exhibit pH‐dependence. This was initially confirmed by observing the color change in the polymer solution at different pH (Figure S12). Upon acidification, the polymer solution displayed a deeper blue color, corresponding to the enhanced absorbance around 650 nm (Figure 2a,b). This shift may be due to the change of the protonation state of the secondary amines linking IR780 to the polymer.^[^ ^28^ ^]^ Also, under 650 nm excitation, the polymer exhibited weak fluorescence emission at pH 7.4, likely due to the intramolecular quenching (Figure S13). However, the fluorescence of the polymer in pH 4.5 buffer was notably higher than that observed at pH 7.4.

To further understand the pH‐dependent properties, the electron distribution in the frontier molecular orbitals of the polymer under different protonation states was analyzed by density functional theory (DFT) computation (Figure 2c). The results confirmed that the energy levels of the highest occupied molecular orbital (HOMO) and the lowest unoccupied molecular orbital (LUMO) differed between the protonated and deprotonated states. Specifically, the HOMO–LUMO energy gap (Δ*E*) was reduced in the protonated state (1.89 eV) compared to de‐protonated state (2.15 eV).

Considering that a lower Δ*E* can enhance the nonradiation decay rate of the excited molecule,^[^ ^29^ ^]^ we evaluated the photothermal and photodynamic activity of LITS under different pH conditions. Clearly, LITS demonstrated a higher photothermal conversion and ROS generation at pH 4.5 compared to pH 7.4 (Figure 2d–f). Because ROS may damage nucleic acids,^[^ ^30^ ^]^ the integrity of the mRNA loaded in the LITS was also investigated after irradiation. The results showed no detectable mRNA degradation under short irradiation time (≤3 min) (Figure 2g). Importantly, the protonation of the secondary amine also alters the charge of the polymer, disrupting the electrostatic balance between polymer and mRNA, which can trigger the dissociation of LITS. To confirm this, the stability of LITS in presence of polyanion was tested under different pH by FCS (Figure 2h). The LITS at pH 4.5 exhibited a diffusion coefficient higher than that at pH 7.4 even in the absence of dextran sulphate (*S/P* = 0), indicating the destabilization of the particles in acidic environment. At pH 4.5 and *S/P* = 4, the LITS achieved a diffusion coefficient equivalent to that of free mRNA, which corresponds to a complete release of mRNA. In contrast, the LITS at pH 7.4 maintained a low diffusion coefficient across all measured *S/P* values. These findings indicate that LITS can sense the acidic pH of endosomal environment to facilitate mRNA release, while simultaneously activating the photosensitizing effects of the polymer without causing mRNA damage.

### Light Accelerates Endosomal Escape to Enhance mRNA Transfection

We first established the experimental conditions to achieve safe and efficient endosomal escape of LITS. The cellular uptake kinetics of LITS were assessed in CT26 cells by confocal laser scanning microscopy (CLSM) with LITS loading Cy5‐labeled mRNA (Figure S14). LITS showed higher cellular uptake than PIC, probably due to its enhanced stability. Moreover, the cellular uptake of LITS reached a plateau after 6 h. Thus, a 6 h incubation of the LITS with cells was selected before initiating irradiation in the subsequent experiments to ensure a sufficient internalization of LITS into cells. We also confirmed that PEG‐pLL(IR780) was not cytotoxic in dark conditions, maintaining cell viability above 90% even at a high concentration of 0.5 mg mL^−1^ (Figure S15), which is much higher than the concentrations used for cellular transfection. Moreover, to avoid potential light‐induced ROS accumulation and cytotoxicity from prolonged irradiation of the photosensitizing polymer (Figures S16 and S17), the irradiation time and polymer concentration were screened to determine the safe irradiation time window that does not decrease cell viability (Figure S18). An irradiation time of 1.5 min was identified to be the maximum time capable of maintaining cell viability above 90% across all tested polymer concentrations. Therefore, this condition was chosen for the following investigations.

**Figure 3:**
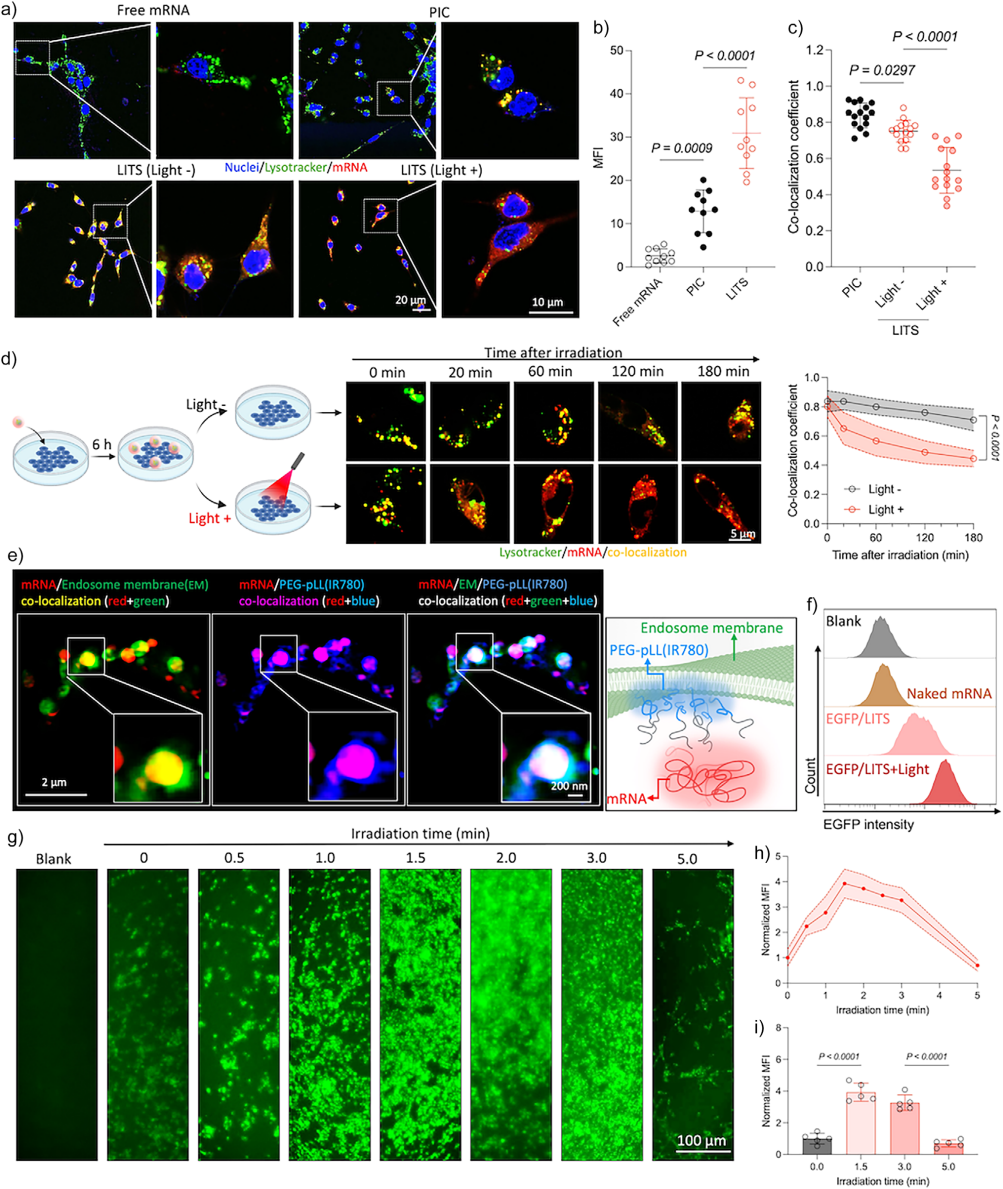
Light accelerates endosomal escape to enhance mRNA transfection. a) Representative microscopic images showing the mRNA delivery efficiency in CT26 cells by different systems. Blue: Hoechst‐stained nuclei, green: Lysotracker‐stained endosome, red: Cy5‐labeled mRNA. b) Mean fluorescence intensity (MFI) of red channel (Cy5‐labeled mRNA) from the cells in the images of (a). Data are plotted as the mean ± S.D., *n *= 10 independent images. The results were compared via one‐way ANOVA. c) The Pearson's co‐localization coefficient of the red pixels (Cy5‐labeled mRNA) to the green pixels (Lysotracker‐stained endosome) in the cells presented in (a). Data are plotted as the mean ± S.D., *n *= 15 independent cells. The results were compared via one‐way ANOVA. d) Endosomal escape kinetics of mRNA delivered by LITS with or without light irradiation. The cells were incubated with LITS for 6 h, then irradiated by a laser. The subcellular distribution of mRNA was tracked by confocal microscopic imaging. The Pearson's co‐localization coefficients of red (Cy5‐labeled mRNA) to green (Lysotracker‐stained endosome) pixels were quantified to indicate the endosomal escape efficiency. Data are plotted as the mean ± S.D., *n *= 10 independent images. The values at 180 min were compared via student's *t*‐test. e) Representative super‐resolution microscopic images and schematic illustration showing the subcellular localization of mRNA and PEG‐pLL(IR780) green: GFP‐labeled endosome membrane, blue: A405‐labeled PEG‐pLL(IR780), red: Cy5‐labeled mRNA, yellow: co‐localization of green and red pixels, pink: co‐localization of blue and red pixels, white: co‐localization of blue, green, and red pixels. f) Representative flow cytometry results showing the EGFP fluorescence intensity from the cells after different treatment. g) Representative fluorescence microscopic images of EGFP/LITS‐treated CT26 cells after irradiation with different time. h) and i) Quantification of the green fluorescence from the images in g) Data are plotted as the mean ± S.D., *n *= 5 independent images. The results were compared via one‐way ANOVA.

The impact of irradiation on the endosomal escape capability of LITS was then examined by CLSM using Cy5‐labeled mRNA. The cells were incubated with free mRNA, PIC and LITS for 6 h. Next, some cells incubated with LITS were irradiated for 1.5 min, then incubated for another 3 h. Comparing to free mRNA and PIC, LITS delivered more mRNA into the cells (Figure 3a,b), and decreased the co‐localization of mRNA with the endosomes (Figure 3c). Furthermore, light irradiation significantly expanded the endosomal escape of the mRNA delivered by LITS. The dynamic time lapse profile of endosome escape after light irradiation was then followed by CLSM. The results showed that light irradiation accelerated the endosomal escape with the co‐localization of LITS and the endosomes rapidly decreasing 25% in 20 min (Figure 3d). After this initial phase, the rate of endosomal escape slowed down, eventually becoming comparable to that of nonirradiated LITS. These findings indicate light irradiation induces an abrupt destabilization of the endosomal membrane to facilitate the access of mRNA into the cytosol.

To further understand the mechanism of endosomal escape, we investigated the subcellular localization of LITS using super‐resolution microscopic imaging and labelling the endosomal membranes with Rab5a‐GFP (Figure 3e). Following cellular uptake, both the polymer (blue) and mRNA (red) were detected within endosomes (green), confirming that the LITS entered cells via the endocytosis pathway.^[^ ^31^ ^]^ Notably, the mRNA and polymer exhibited distinct localization patterns. mRNA was predominantly confined to the endosome lumen, whereas the polymer was able to co‐localize with the endosomal membrane. These findings suggest that at the acidic endosomal environment the LITS releases polymers that interact with the endosomal membrane. This process likely facilitates the subsequent endosome rupture via light‐induced hyperthermia and ROS generation.^[^ ^20^ ^]^

To evaluate the protein translation after irradiation, we used a model mRNA encoding enhanced green fluorescent protein (EGFP). Compared to naked EGFP mRNA and PIC, LITS loading EGFP mRNA (EGFP/LITS) induced significantly higher EGFP expression (Figures 3f and S19), which was further boosted after irradiation (Figure 3f–i). Extending the irradiation to 5 min decreased the EGFP expression level (Figure 3g–i) due to the loss of cell viability. In addition, we treated the cells with the ROS inhibitor *N*‐acetyl‐*L*‐cysteine (NAC) to investigate the roles of ROS and hyperthermia induced by irradiation in protein translation. The results showed that NAC partially reduced the transfection efficiency (Figure S20), suggesting both the ROS and the hyperthermia generated by the photosensitizing polymer contributed to the light‐induced transfection.

### LITS Controls Spatiotemporal mRNA Transfection in Vivo

**Figure 4:**
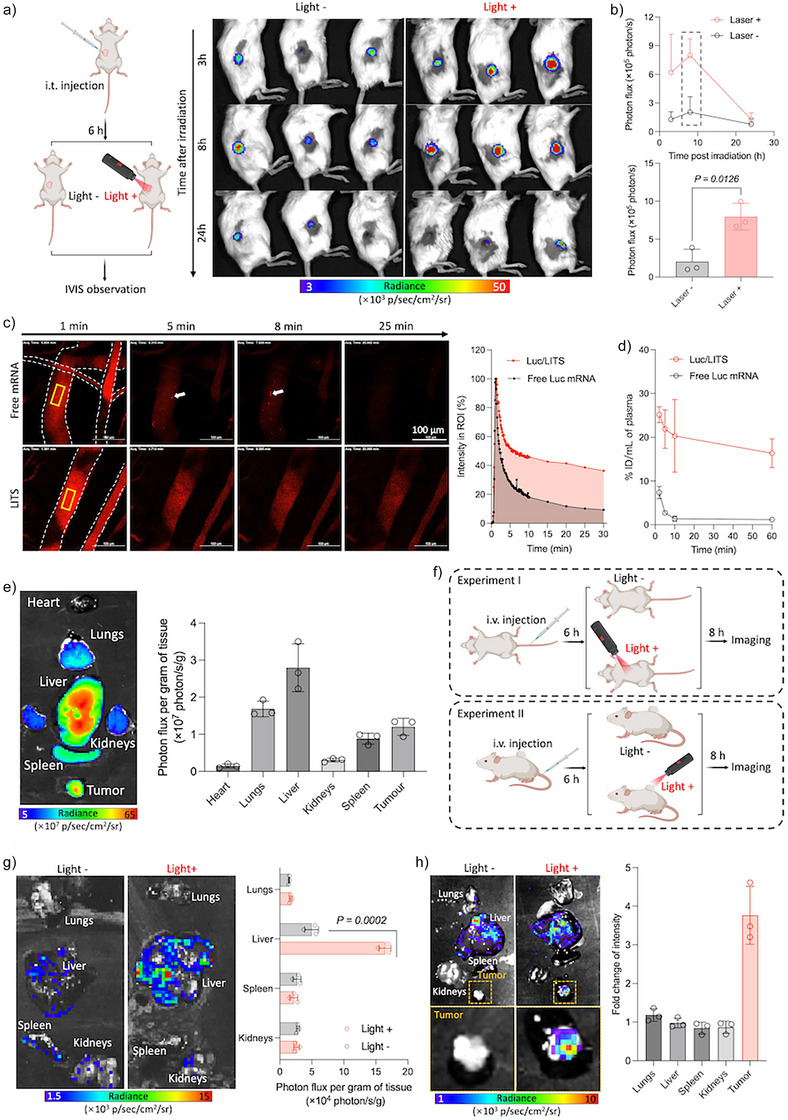
LITS controls spatiotemporal mRNA transfection in vivo. a) Intratumoral transfection efficiency of LITS upon i.t. injection. Luc/LITS was i.t. injected to mice bearing CT26 tumors. Irradiation was conducted at 6 h post injection. At different time (3‐, 8‐, and 24‐ h) after irradiation, the mice were imaged by IVIS to detect the bioluminescence signals from the tumors. b) Quantification of radiance intensities from the tumors. The time‐dependent profile was plotted at the upper right panel. The individual radiance intensity from the tumors at 8 h post irradiation was plotted at the lower right panel. Data are plotted as the mean ± S.D., *n *= 3 independent animals. The results were compared via one‐way ANOVA. c) IVCLSM images showing the circulation of Cy5‐labeled mRNA (red) in the vessels (area indicated by the dashed boarder) on mice earlobe upon i.v. injection. The white arrow indicates the aggregations formed in blood stream after injection of free mRNA. The fluorescence intensity profiles were measured from the ROIs (yellow boxes) and plotted at the right panel. d) Quantitative mRNA circulation profile in blood measured by qPCR. Data are plotted as the mean ± S.D., *n *= 3 independent samples. e) Representative IVIS fluorescence image showing the distribution of mRNA in main organs and tumor at 6 h after i.v. injection of LITS. The intensities in the tissue samples were quantified and plotted at the right panel. f) Schematic illustration of the experiments investigating the mRNA delivery outcomes by LITS upon i.v. injection. Mice were i.v. injected with Luc/LITS. Irradiation was conducted at 6 h post injection at the liver area (Experiment I) or tumor area (Experiment II). At 8 h after irradiation, tissue samples were imaged by IVIS to detect the bioluminescence signals. g) Representative IVIS bioluminescence image showing the Luc expression in the organs in Experiment I. The intensities were quantified and plotted at the right panel. Data are plotted as the mean ± S.D., *n *= 3 independent animals. The results were compared via Student's *t*‐test. h) Representative IVIS bioluminescence image showing the Luc expression in the tissue samples in Experiment II. The intensity changes in the organs and tumors of the irradiated mice over the nonirradiated mice were quantified and plotted at the right panel. Data are plotted as the mean ± S.D., *n *= 3 independent animals.

The in vivo performance of LITS was evaluated in mice, with the initial investigation focusing on intratumoral (i.t.) injection as a local administration route. Thus, LITS loaded with luciferase (Luc)‐encoding mRNA (Luc/LITS) was injected into CT26 tumors. 6 h after injection, a 1.5 min laser irradiation was applied to the tumor area. The expression level of Luc in the tumor was measured using bioluminescence via intravital imaging system (IVIS) (Figure 4a,b). Luc/LITS successfully transfected the tumors. The bioluminescence signal peaked at 8 h post‐irradiation, with irradiated tumors displaying around 4‐fold higher signal intensity compared to the nonirradiated group. These findings confirm the capability of LITS to boost the local protein expression with light irradiation.

LITS was then tested for intravenous (i.v.) injection applications. The blood circulation of LITS was analyzed using intravital CLSM (IVCLSM) using Cy5‐labeled Luc mRNA (Figure 4c; Videos S4 and S5). Shortly after injection, free mRNA exhibited aggregates with micrometer size, consistent with previous reports.^[^ ^32^, ^33^ ^]^ In contrast, LITS circulated stably in the vessels without aggregation. Quantitative analysis of the fluorescence intensity in blood vessels revealed longer circulation for LITS compared to free mRNA. Furthermore, RT‐PCR analysis showed that LITS preserved the integrity of mRNA in the bloodstream, whereas free mRNA rapidly degraded after injection (Figure 4d). Following i.v. injection, LITS delivered mRNA to several major organs, including the liver, spleen, lungs, and kidneys (Figure 4e). Notably, significant accumulation of the mRNA in LITS was detected in tumors.

Considering the ability of LITS to deliver mRNA into different tissues, we then investigated the light‐induced transfection efficiency in different tissues after i.v. administration of Luc/LITS (Figure 4f). Since liver exhibited the highest mRNA accumulation, we first evaluated the effect of irradiation on the transfection efficacy in the liver of healthy mice using Luc mRNA. 6 h after i.v. injection of Luc/LITS, the liver area was externally irradiated for 1.5 min. The tissues were harvested 8 h later for bioluminescence imaging to assess the Luc expression. Comparing to nonirradiated mice, the irradiated mice displayed a stronger (∼3.5‐fold) local signal in the liver (Figure 4g), confirming that irradiation boosted the mRNA transfection in a light‐controlled manner.

The ability of systemically injected LITS to transfect CT26 tumors was then investigated (Figure 4h). The tumors were irradiated for 1.5 min 6 h after i.v. injection of Luc/LITS. Bioluminescence imaging of the tissue samples was performed 8 h later. While the bioluminescence signals from organs were comparable between the irradiated and nonirradiated mice, the signals in tumors increased by approximately 3.5‐fold upon light irradiation (Figure 4h), and the irradiated tumors exhibited higher bioluminescence intensity compared to all the organs, including the liver (Figure S21). A similar trend was observed with EGFP/LITS (Figure S22), where EGFP expression in tumor was markedly increased after irradiation. These results demonstrate the ability of LITS to achieve a precise local boost of mRNA transfection in tissues through light irradiation.

### LITS Controls the Pleiotropic Immune Signals of IL‐2

**Figure 5:**
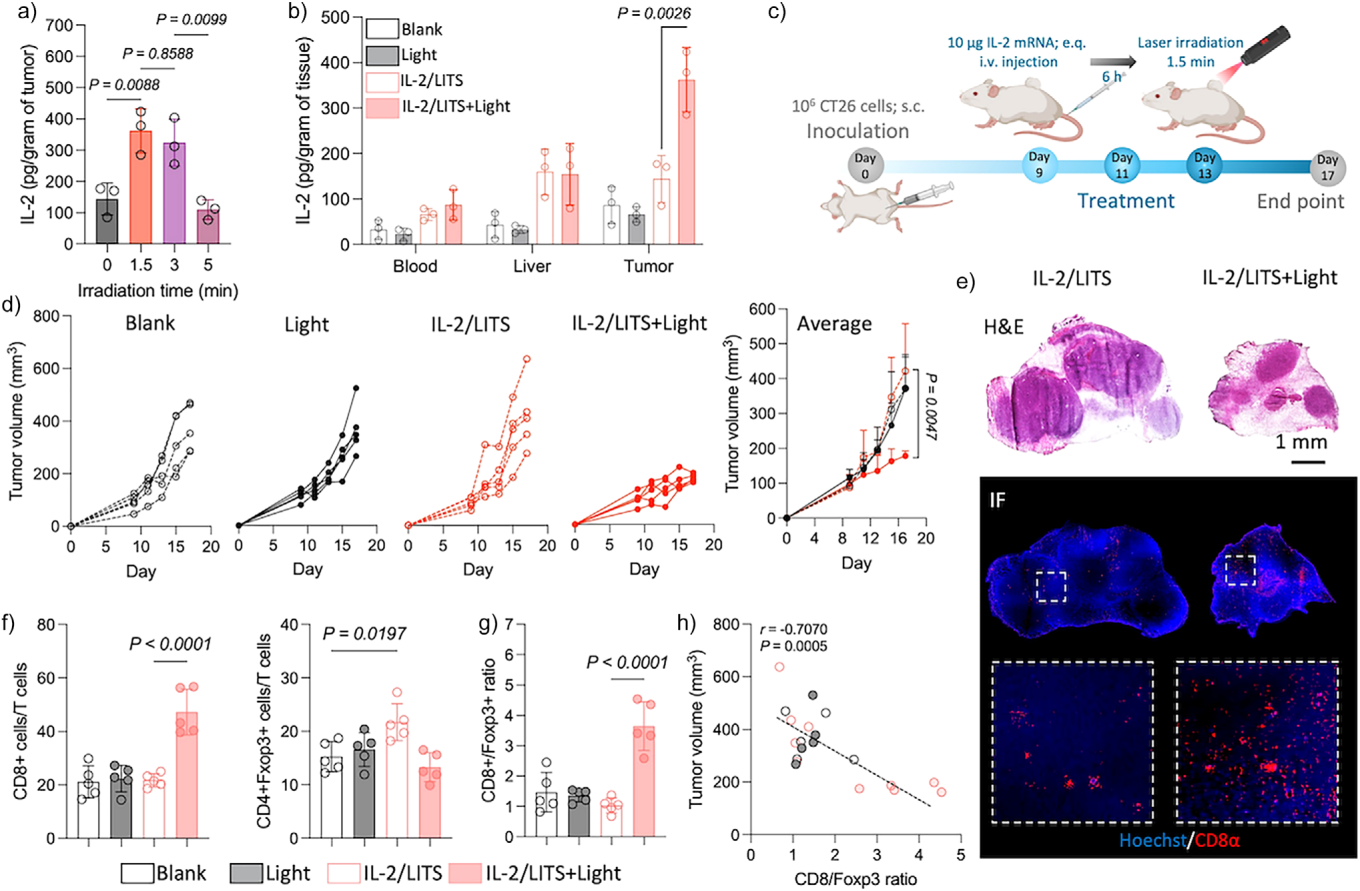
IL‐2/LITS potentiates antitumoral immunity upon irradiation. a) IL‐2 expression level in the tumors with different irradiation time after administration of IL‐2/LITS. Data are plotted as the mean ± S.D., *n *= 3 independent samples. The results were compared via one‐way ANOVA. b) In vivo expression of IL‐2 in tissue samples of mice bearing CT26 tumors after treatments. Data are plotted as the mean ± S.D., *n *= 3 independent animals. c) Schematic illustration showing the schedule of the following experiment. Mice were s.c. inoculated with CT26 cells on day 0. Three treatments were conducted on days 9, 11, and 13. Mice were scarified on day 17 for immune analysis. d) Tumor growth during the experiment. The tumor volume on day 17 were compared among different groups. Data are plotted as the mean ± S.D., *n *= 5 independent animals. e) Representative histological analysis of tumor sections harvested on day 17. The samples were stained by H and E staining and fluorescence‐labeled anti‐CD8α. f) The populations of CD8+CTL and Foxp3+Tregs in tumor‐infiltrating T cells measured on day 17 by flow cytometry. Data are plotted as the mean ± S.D., *n *= 5 independent animals. g) CD8/Foxp3 ratio in the tumors on day 17. Data are plotted as the mean ± S.D., *n *= 5 independent animals. h) Correlation between the final tumor size and CD8/Foxp3 ratio evaluated via Pearson correlation analysis. In this figure, all results were compared via one‐way ANOVA.

To demonstrate the therapeutic potential of LITS, we selected IL‐2 mRNA, as this cytokine exhibits dose‐dependent pleiotropic effects,^[^ ^34^ ^]^ requiring precise spatial regulation throughout the body to achieve safe and effective therapeutic outcomes. The performance of LITS loaded with mRNA encoding IL‐2 (IL‐2/LITS) was first evaluated in CT26 cells in vitro (Figure S23). Following 1.5 min irradiation, IL‐2/LITS induced the highest IL‐2 expression. In vivo, i.v. injected IL‐2/LITS also demonstrated its ability to produce IL‐2 in CT26 tumors (Figure 5a). Consistent with the in vitro findings, an irradiation of 1.5 min resulted in the highest intratumoral IL‐2 levels. Thus, 1.5 min irradiation was selected for the following in vivo experiments. After systemic administration, IL‐2/LITS induced high IL‐2 levels in irradiated tumors, while maintaining moderate IL‐2 levels in blood, liver, and nonirradiated tumors (Figure 5b).

As the high IL‐2 levels may promote antitumor immunity, we studied the antitumoral efficacy of IL‐2/LITS in the CT26 tumor model (Figure 5c). 6 h after i.v. injection of IL‐2/LITS, the tumors were irradiated for 1.5 min. This treatment was repeated three times every 2 days. The tumors in the group receiving both IL‐2/LITS and irradiation (IL‐2/LITS+Light) exhibited significantly slower tumor growth compared to the other groups, including untreated mice (Blank), mice receiving only irradiation (Light), and nonirradiated mice injected with IL‐2/LITS (Figure 5d). Such antitumoral efficacy should be attributed to the priming of the tumor microenvironment (TME), as indicated by the differentiated cytokine levels in tumors after the treatments (Figure S24). Specifically, IL‐2/LITS+Light upregulated proinflammatory cytokines, including IFN‐γ, IL‐1β, and IL‐6, contributing to a shift of the TME toward a proinflammatory phenotype.^[^ ^35^, ^36^, ^37^, ^38^ ^]^ In contrast, the other treatments failed to promote proinflammatory cytokines in the tumor. Particularly, IL‐2/LITS without irradiation increase the level of anti‐inflammatory IL‐10 in tumors, suggesting the occurrence of immunosuppression by the moderate IL‐2 expression.^[^ ^39^ ^]^

The presence of the cytokines in the TME changed the intratumoral immune infiltration landscape (Figures 5e and S25). After IL‐2/LITS+Light treatment, CD8+cytotoxic T lymphocytes (CTL) were expanded in the tumor (Figure 5e,f), while Foxp3+Tregs showed moderate change compared to control group. Notably, IL‐2/LITS without irradiation increased the Treg population in the tumor, which could be associated with the high expression of the IL‐2 receptor alpha chain (CD25) on Tregs, allowing them to respond to even small amounts of IL‐2.^[^ ^40^ ^]^ Moreover, IL‐2/LITS+Light treatment showed the highest ratio between CD8+T cells and Foxp3+Tregs (CD8/Foxp3), which is a biomarker supporting the antitumoral immune response^[^ ^41^, ^42^ ^]^ (Figure 5g). Indeed, the final tumor size and the CD8/Foxp3 ratio showed high correlation (*r* = −0.707, *p* = 0.0005) (Figure 5h). These results show that irradiated IL‐2/LITS can successfully drive the proinflammatory signals of IL‐2 for evoking an antitumoral immune response in the TME.

**Figure 6:**
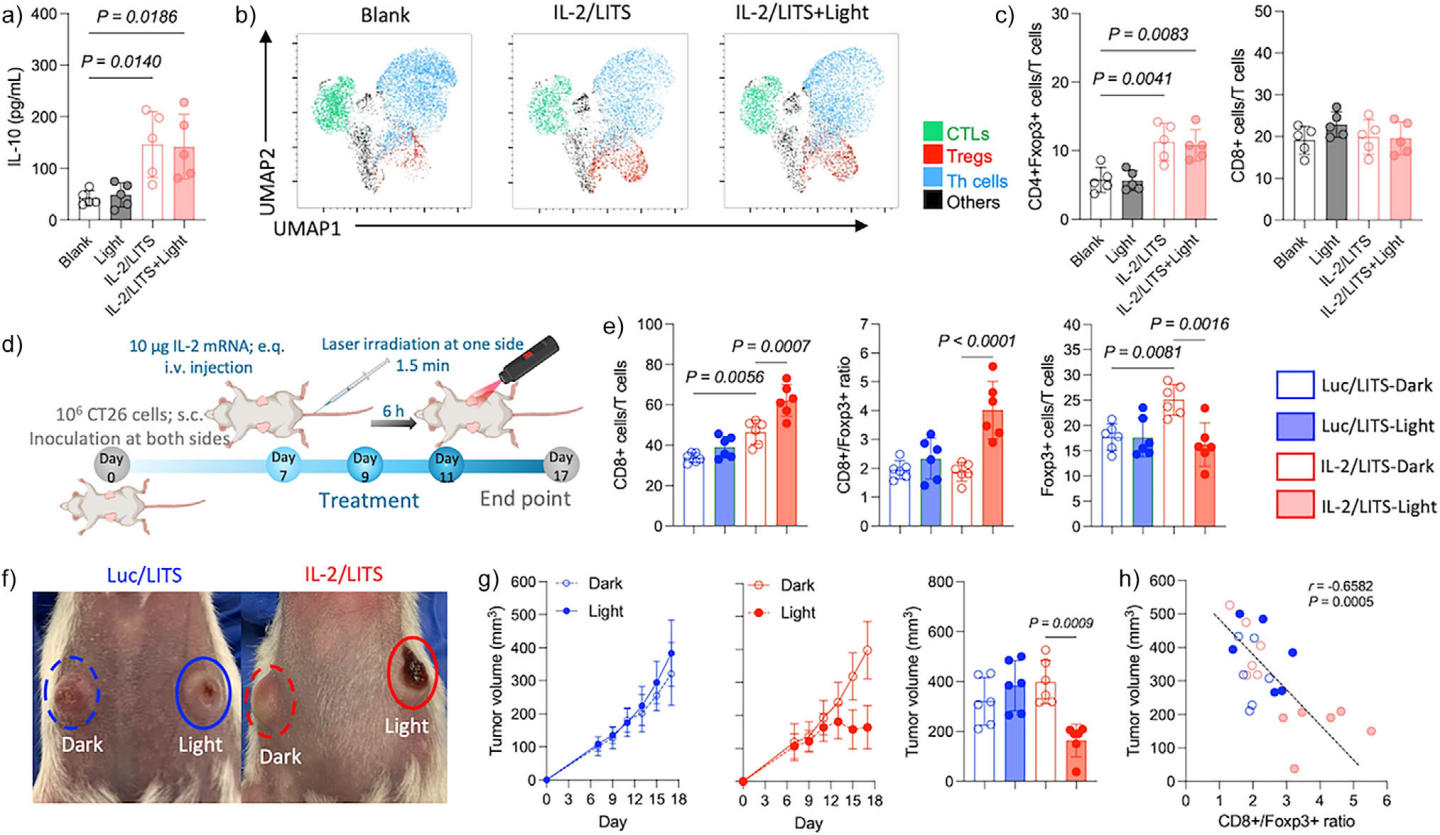
IL‐2/LITS elicit protective immunosuppressive signals systemically. a) Plasma IL‐10 concentration on day 17. Data are plotted as the mean ± S.D., *n *= 5 independent animals. b) Uniform manifold approximation and projection (UMAP) dimension reduction analysis of flow cytometry results showing the T cell sub‐population in blood on day 17. c) The populations of Foxp3+Tregs and CD8+CTLs in blood T cells measured on day 17. Data are plotted as the mean ± S.D., *n *= 5 independent animals. d) Schematic illustration showing the experiment schedule of dual primary tumor model. Mice were s.c. inoculated with CT26 cells on day 0. Three treatments were conducted on day 7, 9, and 11. Mice were scarified on day 17 for immune analysis. e) The populations of CD8+CTLs and Foxp3+Tregs, and the CD8/Foxp3 ratios in the tumors. Data are plotted as the mean ± S.D., *n *= 6 independent tumors. f) Representative images of the tumors on day 12. g) Averaged tumor growth curves during the experiment. Tumor volumes on day 17 were plotted on the right panel. Data are plotted as the mean ± S.D., *n *= 6 independent tumors. h) Correlation between final tumor size and CD8/Foxp3 ratio evaluated via Pearson correlation analysis. In this figure, all results were compared via one‐way ANOVA.

The systemic immune responses in blood, liver, and spleen were also analyzed. The cytokine profiling in blood revealed that the IL‐2/LITS treatment with or without tumor irradiation did not increase the inflammatory cytokines, including IFN‐γ, IL‐1β, and IL‐12 (Figure S26). However, anti‐inflammatory IL‐4 and IL‐10 were upregulated by IL‐2/LITS with or without tumor irradiation (Figures 6a and S26), suggesting the activation of systemic immunosuppressive signals.^[^ ^43^ ^]^ The increase in IL‐4 and IL‐10 is known to support the expansion and function of Tregs,^[^ ^44^, ^45^, ^46^ ^]^ which corresponds with the expansion of Foxp3+Tregs in blood after IL‐2/LITS treatment (Figures 6b,c and S27). On the other hand, the population of CD8+CTLs in blood remained unchanged. Analysis of immune T cell subtypes in the liver and spleen showed no significant differences between control and IL‐2/LITS‐treated groups (Figure S28). These results indicate the IL‐2/LITS avoided systemic CD8+CTL expansion and Treg suppression, which are commonly observed in high dose IL‐2 treatments and related with immune toxicity.^[^ ^47^ ^]^ Indeed, the safety of the treatments was confirmed by the analysis of blood toxicity markers and histological observation, which showed no difference across all groups (Figures S29 and S30).

To directly compare the pro‐ and anti‐inflammatory signaling pathways induced by IL‐2/LITS, we studied the antitumor effects of IL‐2/LITS in a dual primary tumor model by inoculating CT26 cells on both sides of the abdomen (Figure 6d). IL‐2/LITS or Luc/LITS were i.v. injected, but the irradiation was restricted to only one tumor per mouse. Following IL‐2/LITS treatment, the irradiated tumors exhibited a high intratumoral IL‐2 concentration, successfully triggering IFN‐γ dependent inflammation (Figure S31). In contrast, the nonirradiated tumors showed increased IL‐10 levels within the TME. This differentiation in cytokine signaling led to contrasting intratumoral immune infiltration patterns within the same mouse. Irradiated tumors displayed robust infiltration by CD8+CTLs, while nonirradiated tumors were characterized by an upregulation of Foxp3+Tregs (Figures 6e and S32). These results demonstrate that the irradiation spatially confined the proinflammatory effects of IL‐2 to the treated tumor, while nonirradiated tumors remained dominated by the anti‐inflammatory signaling. This contrasting immune modulation in tumors resulted in distinct tumor progression (Figure 6f), where only the growth of the tumors at the irradiated side was suppressed (Figure 6g). Additionally, a strong correlation was observed between the final tumor volumes and the CD8/Foxp3 ratio within each tumor (Figure 6h), confirming the robust capability of IL‐2/LITS to spatiotemporally control the pleiotropic effects of IL‐2.

### IL‐2/LITS Synergizes with Phototherapy to Eradicate Tumors

**Figure 7:**
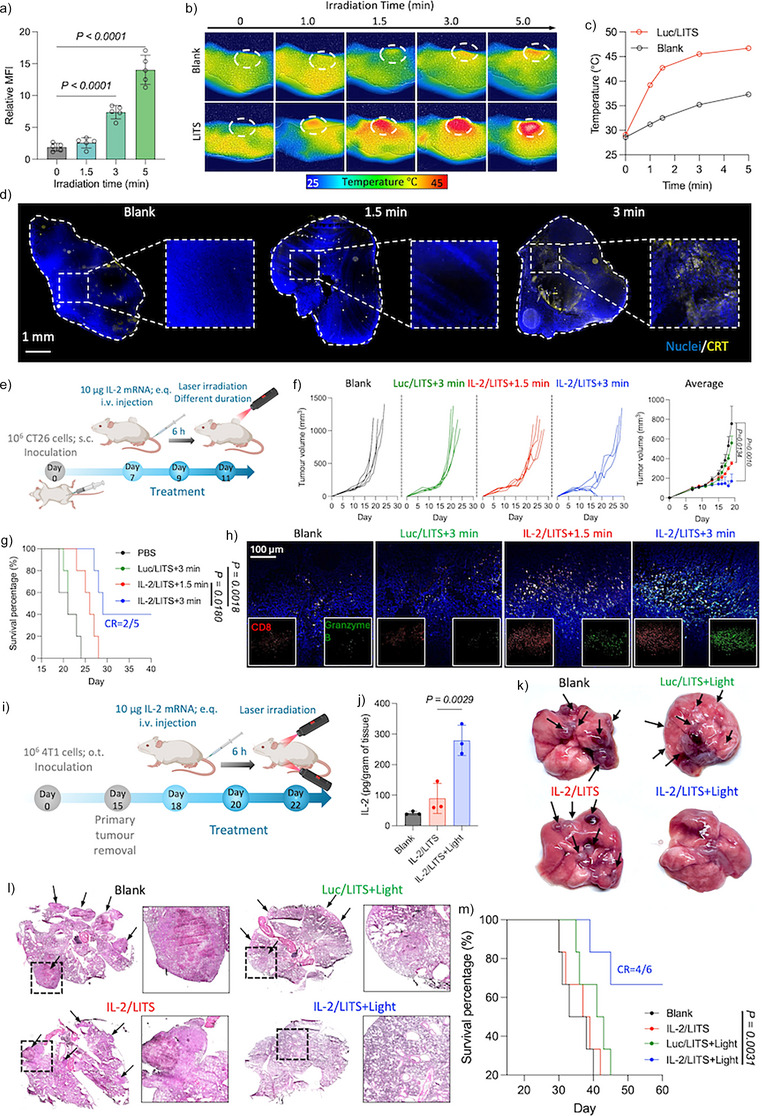
IL‐2/LITS synergizes with phototherapy for tumor regression. a) Expression level of calreticulin (CRT) on CT26 cells upon different irradiation time after Luc/LITS treatment. Data are plotted as the mean ± S.D., *n *= 5 independent images. The results were compared via one‐way ANOVA. b) Thermal images of mice bearing CT26 tumors (white circle) irradiated after administration of Luc/LITS. c) The temperature of tumor area upon different irradiation time. d) Representative immunostaining images of tumor sections with different irradiation time after administration of Luc/LITS. e) Schematic illustration showing the schedule of the experiment evaluating the performance of IL‐2/LITS in CT26 primary tumors with different irradiation time. Mice were s.c. inoculated with CT26 cells on day 0. Three treatments were conducted on days 7, 9, and 11. Mice were kept until death or the tumor volume exceeded 1000 mm^3^. f) Tumor growth curves during the experiment. Individual curves of each group were plotted on the left panel. The tumor volumes in each group were averaged and plotted on the right panel. Data are plotted as the mean ± S.D. (*n *= 5 independent animals). Tumor volumes on day 19 (when the first death case occurred) were compared among different groups via one‐way ANOVA. g) Survival curves of the animals during the experiment. The results were compared via the Log‐rank test. h) Representative immunofluorescence staining of tumor sections on day 20. i) Schematic illustration showing the schedule of the experiment evaluating the performance of IL‐2/LITS in 4T1 lung metastasis model. Mice were orthotopically (o.t.) inoculated with 4T1 cells on day 0. The primary tumors were removed surgically on day 15. Treatments were administered on days 18, 20, and 22. j) IL‐2 levels in the lungs measured 8 h after the first treatment. Data are plotted as the mean ± S.D., *n *= 3 independent samples. The results were compared via one‐way ANOVA. k) Photographs of representative lung samples from each treatment group on day 30. l) Representative H and E staining of the lung sections on day 30. m) Survival curves of the animals during the experiment. *n* = 6 independent mice. The results were compared via the Log‐rank test.

LITS generates ROS and hyperthermia that may directly induce damage of cells, thereby achieving photodynamic and photothermal therapies (PDT and PTT, respectively).^[^ ^48^, ^49^ ^]^ PDT and PTT have also been reported to trigger ICD,^[^ ^50^ ^]^ which can synergize with immunotherapies to enhance efficacy. Luc/LITS treated CT26 cells showed increased expression of the ICD marker calreticulin (CRT) after extending the irradiation time (Figures 7a and S33),^[^ ^51^ ^]^ confirming the potential of LITS to induce ICD. Moreover, i.v. injected Luc/LITS effectively heated the CT26 tumors when irradiated (Figure 7b). Notably, just 3 min irradiation was sufficient to raise tumor temperature above 42 °C (Figure 7c), which can initiate the PTT process.^[^ ^52^, ^53^, ^54^ ^]^ Histological analysis revealed that 3 min irradiation after Luc/LITS treatment upregulated CRT in tumor tissues (Figure 7d), and induced apoptosis/necrosis (Figure S34). Considering that IL‐2/LITS also provides a high IL‐2 expression level within tumors after 3 min irradiation (Figure 5a), we hypothesized that the cooperation between IL‐2 and ICD could lead to synergistic therapeutic effects. To validate this, we evaluated the efficacy of IL‐2/LITS with different irradiation time in the CT26 tumor model (Figure 7e). The combination of IL‐2/LITS treatment with light irradiation clearly inhibited tumor growth (Figure 7f). Notably, with IL‐2/LITS treatment, extending the irradiation time to 3 min significantly enhanced therapeutic efficacy compared to 1.5 min (Figure 7f), which prolonged the survival of the animals with two out of five mice achieving complete responses (CR) (Figure 7g). Immunostaining analysis revealed that IL‐2/LITS treatment with 3 min irradiation induced the highest infiltration of CD8+CTLs in CT26 tumors (Figure 7h) and significantly elevated Granzyme B level in these CTLs.^[^ ^55^, ^56^ ^]^ As a result, IL‐2/LITS with 3 min irradiation demonstrated the strongest tumor‐damaging effects (Figure S35). Meanwhile, no significant toxicity was observed during the experiment, with all animals presenting similar bodyweight across all groups (Figure S36).

Beyond subcutaneous tumor models, the optoregulated tissue‐targeting capability of LITS enables precise treatment of disseminated tumor lesions, as shown in a lung metastasis model of triple negative breast cancer (TNBC) (Figure 7i). Upon i.v. injection of IL‐2/LITS, irradiation was applied to the chest front and back areas of the mice. This treatment successfully evoked IL‐2 expression in lungs (Figure 7j). The IL‐2/LITS+Light treatment showed effective inhibition of the lung metastases, as illustrated by macroscopic (Figure 7k) and H and E histological observations (Figure 7l). Moreover, the IL‐2/LITS+Light treatment demonstrated the longest survival of the animals, achieving CR in four out of six mice in the group (Figure 7m). In contrast, the other treatments, including IL‐2/LITS without light and Luc/LITS plus Light, failed to elicit significant survival benefits. Meanwhile, all the mice showed comparable bodyweight during the experiment (Figure S37), indicating the safety of the treatment.

## Conclusion

Our findings demonstrate that a strategically designed light‐activated delivery platform can address one of the fundamental challenges in mRNA therapeutics through tissue‐specific expression after systemic administration. By decoupling the delivery and activation steps, LITS allows overcoming the traditional trade‐off between broad distribution and targeted action that has long constrained the development of mRNA therapeutics. This light‐controlled activation was particularly evident in our IL‐2 model, where we could selectively induced proinflammatory cytokine levels in irradiated tumors, while maintaining immune tolerance in healthy tissues.

Equipping LITS with the IR780 photosensitizer was critical for achieving systemic delivery and precise optoregulation in vivo. The positive net charge, hydrophobicity, and planar structure of IR780 facilitated mRNA complexation through multiple weak interactions,^[^ ^57^, ^58^, ^59^ ^]^ enhancing the stability of LITS after intravenous injection, as reflected in its superior pharmacokinetics. Moreover, our results demonstrated a broad biodistribution of LITS, effectively targeting the liver, lungs, and tumors, which is a rare feature among mRNA delivery systems. This performance likely stems from LITS's densely PEGylated surface, which could minimize the formation of a protein corona^[^ ^60^, ^61^ ^]^ (commonly used in other mRNA delivery systems for guiding systemic biodistribution^[^ ^10^, ^12^, ^62^ ^]^) and reduce nonspecific cellular uptake.^[^ ^63^ ^]^ Its sub‐100 nm size may also decrease RES retention^[^ ^64^, ^65^ ^]^ and promote tumor penetration.^[^ ^66^ ^]^ These findings establish LITS as a robust mRNA delivery platform. To further improve the delivery precision, LITS can be integrated with ligand‐directed approaches by improving tissue distribution and cellular uptake.^[^ ^67^ ^]^ In addition, incorporating functional moieties such as cationic amphiphilic drugs (CADs) may further boost delivery efficiency and enable synergistic therapeutic effects.^[^ ^68^ ^]^

The IR780 conjugation approach also generated ionizable secondary amines in the polymer, enabling pH‐responsive mRNA release and photosensitizing activity within the acidic endosomal environment. Upon irradiation, the IR780‐conjugated polymers elicited localized hyperthermia and ROS that were essential for endosomal escape and mRNA translation. Given that several photosensitizers with excitation wavelengths around 650 nm are already used in the clinic,^[^ ^69^ ^]^ the LITS platform demonstrates strong translational potential. Moreover, IR780 is just a model photosensitizer, and the LITS platform can be readily adapted to other photosensitizers with comparable structural properties. This versatility would enable the development of an orthogonal LITS library, where each formulation is tuned to a specific activation wavelength. Such a platform could facilitate multiplexed protein expression, allowing for spatiotemporally coordinated therapeutic interventions. Additionally, employing photosensitizers with excitation wavelengths in the near‐infrared (NIR) range offers deeper tissue penetration and holds greater translational potential compared to that at the current 650 nm wavelength.

The ability of LITS to manipulate cytokine signaling with tissue specificity has the potential to transform immunotherapy by precisely controlling immune activation, while avoiding systemic toxicity.^[^ ^70^, ^71^ ^]^ This principle is exemplified by the IL‐2 system explored in our study, where LITS enable effective control of its pleiotropic effects for safe antitumor effects. Although IL‐2 is used in clinical cancer therapy,^[^ ^72^ ^]^ its application is limited by severe side effects and the activation of Tregs at low dose that jeopardize the desired immune responses.^[^ ^73^, ^74^ ^]^ To enhance its therapeutic potency, structurally modified IL‐2 derivatives have been developed to bias IL‐2 signaling toward proinflammatory pathways.^[^ ^75^, ^76^, ^77^, ^78^ ^]^ However, these approaches overlook the critical role of Tregs in immune tolerance,^[^ ^47^ ^]^ increasing immune‐related toxicities.^[^ ^79^, ^80^ ^]^ Our tissue‐specific optoregulation overcomes the limitations of IL‐2 therapy, intensifying effector immunity within tumors, while preserving immune homeostasis in healthy tissues. Moreover, the antitumor immunity can be further augmented through LITS‐mediated ICD. This approach establishes a framework for improving both the therapeutic window and the potency of diverse immunomodulatory proteins.

Integrating LITS with emerging mRNA designs, such as circular RNA^[^ ^81^ ^]^ or self‐replicating RNA,^[^ ^82^ ^]^ could enable further control over protein expression dynamics. In addition to light, ultrasound may also be suitable for activating mRNA transfection, as recently illustrated for spleen and liver delivery.^[^ ^83^ ^]^ Given that IR780 is also a sonosensitizer,^[^ ^84^, ^85^ ^]^ LITS could be applied for ultrasound‐guided mRNA delivery, enhancing tissue permeability compared to light and expanding the range of targetable tissues. These advances have the potential to impact a wide variety of treatments from cancer immunotherapy to regenerative medicine that require targeted protein expression. Beyond its immediate therapeutic applications, our platform advances the broader field by showcasing how strategic molecular design can separate biodistribution from therapeutic action.

## Supporting Information

The authors have cited additional references within the Supporting Information.^[^ ^86^, ^87^, ^88^, ^89^, ^90^, ^91^, ^92^, ^93^ ^]^

## Conflict of Interests

The authors declare no conflict of interest.

## Supporting information



Supporting Information

Supporting Information

Supporting Information

Supporting Information

Supporting Information

Supporting Information

## Data Availability

The data that support the findings of this study are available from the corresponding author upon reasonable request.
