# Supplementary material for: Optoregulated mRNA Delivery Controls Pleiotropic Immune Signaling for Tumor‐Targeted Therapy
Source: Angew Chem Int Ed Engl. 2025 Sep 2;64(43):e202513610. doi: 10.1002/anie.202513610 (PMC12535396; doi:10.1002/anie.202513610)
Supplement: Supplementary file 1 — Supporting Information [file ANIE-64-e202513610-s004.docx]

**Supplementary information**

**Optoregulated mRNA Delivery Controls Pleiotropic Immune Signaling for Tumor-Targeted Therapy**

Pengwen Chen^1^, Guanghao Hu^1^, Yuki Nakashima^1^, Zhining Xu^2,3^, Takayoshi Watanabe^1^, Soichiro Kondo^1^, Ervin Kovács^2^, Horacio Cabral^1^*

^1^Department of Bioengineering, Graduate School of Engineering, The University of Tokyo, Bunkyo-ku, Tokyo, Japan

^2^Institute of Materials and Environmental Chemistry, HUN-REN Research Centre for Natural Sciences, Budapest, Hungary

^3^Hevesy György PhD School of Chemistry, Eötvös Loránd University, Budapest, Hungary

Correspondence to:
E-mail: [horacio@bmw.t.u-tokyo.ac.jp](mailto:horacio@bmw.t.u-tokyo.ac.jp)

1. **Materials and methods**

*1.1 Materials*

α-methoxy-ω-amino-poly(ethylene glycol) (MeO-PEG-NH_2_; molecular weight (Mw) = 12,000 g mol^−1^) was purchased from NOF Corporation (Tokyo, Japan). *N*-trifluoroacetyl-L-lysine *N*-carboxyanhydride (Lys(TFA)-NCA) (purity > 98%) was purchased from Chuo Kasei Co., Ltd (Osaka, Tokyo). IR780 iodide was purchased from Sigma-Aldrich (Burlington, USA). Triethylamine (TEA), and 1,3-diphenylisobenzofuran (DPBF) were purchased from Tokyo Chemical Industry Co., Ltd (Tokyo, Japan). Dimethylformamide (DMF), N-acetyl-L-cysteine (NAC) were purchased from Fujifilm Corporation (Tokyo, Japan). Enhanced Green Fluorescence Protein (EGFP) mRNA and Firefly Luciferase (Luc) mRNA were purchased from TriLink Bio Technologies (San Diego, USA). IL-2 mRNA was purchased from VectorBuilder (Chicago, USA). mRNAs were labeled by Cy5 fluorescence dye by Label IT Nucleic Acid Labelling Kit from Mirus Bio (Madison, USA). Agarose, Qubit RNA HS assay, Lysotracker Green, Alexa 405-NHS ester, calcein/EthD-1 staining kit, CellLight Early Endosomes-GFP, H_2_DCFDA probe, Red Blood Cell Lysis Buffer, collagenase, Live/Dead fixable near-IR dead cell staining dye, Alexa 647-anti Foxp3 (clone 3G3), PerCp-eFluor710-anti-mouse CD8 (clone 53-6.7), Foxp3/transcription factor staining buffer kit, and ELISA kits were purchased from Thermo Fisher (Waltham, USA). Midori Green staining reagent was purchased from Nippon Genetics (Tokyo, Japan). RNeasy Mini kit was purchased from Qiagen (Hilden, Germany). ReverTra Ace qPCR RT Master Mix kit was purchased from Toyobo (Osaka, Japan). TaqMan Universal PCR Master Mix was purchased from Applied Biosystems (Foster City, USA). The primers and probe for qPCR experiments detecting Luc mRNA were purchased from Integrated DNA Technologies (Coralville, USA), and the sequences are as follows: forward primer GTGGTGTGCAGCGAGAATAG, reverse primer CGCTCGTTGTAGATGTCGTTAG, probe TTGCAGTTCTTCATGCCCGTGTTG. Dextran sulphate sodium salt (M_W_ = 40,000) was purchased from Biosynth (Staad, Switzerland). Cell Counting Kit (CCK)-8 and Hoechst 33342 was purchase from Donjindo (Tokyo, Japan). Luciferin was purchase from Promega (Madison, USA). Protease inhibitor cocktail was purchased from Merck (Darmstadt, Germany). PE-anti-mouse CD3 (clone 17A2), Alexa 647-anti-mouse CD8α (clone 53-6.7), and FITC-anti-mouse/human Granzyme B (GB11) were purchased from BioLegend (San Diego, USA). PE-Cy7-anti-mouse CD45 (clone 30-F11), FITC-anti mouse CD4 (clone RM4-5) and anti-mouse CD16/32 (clone 2.4G2) were purchased from BD Biosciences (Franklin Lakes, USA). Hematoxylin and Eosin (H&E) staining kit and (Terminal deoxynucleotidyl transferase dUTP nick end labelling) TUNEL assay kit were purchased from Abcam (Cambridge, UK). Alexa 488-anti-calreticulin (clone D3E6) was purchased from Cell Signaling Technology (Danvers, USA).

*1.2 Cells and animals*

Murine colon carcinoma cell line CT26 and murine triple negative breast cancer cell line 4T1 were purchase from Cell Bank, Riken BioResource Center. CT26 and 4T1 cells were cultured in RPMI-1640 medium (Gibco) supplemented with 10% fetal bovine serum (Biosera), 100 U mL^-1^ penicillin and 100 U mL^-1^ streptomycin (Gibco). Cells were incubated at 37℃ with 5% CO_2_ atmosphere. Female BALB/c mice were purchased from Charles River Laboratory. All animal experiments in this study were approved by The University of Tokyo (A2023E035) and conducted under the Guidelines for the Care and Use of Laboratory Animals.

*1.3 Synthesis and characterization of PEG-pLL(IR780)*

PEG-pLL was firstly synthesized *via* ring opening polymerization (ROP) of Lys(TFA)-NCA using MeO-PEG-NH_2_ as the initiator^[1]^. MeO-PEG-NH_2_ (500 mg) was dissolved in 10 mL 50 mM NaHCO_3_ buffer (pH = 8.4). Lys(TFA)-NCA (1,000 mg) was added to the MeO-PEG-NH_2_ solution in ice bath with fast stirring. The mixture was kept reacting for 12 h, followed by dialysis (membrane molecular weight cut-off (MWCO) = 6,000-8,000) against pure water. The solvent was evaporated under vacuum and the solid was redissolved in methanol and precipitated against diethyl ether to get poly(ethylene glycol)-poly(trifluoroacetyl-L-Lysine) (PEG-pLL(TFA)). The TFA groups were removed by dissolving PEG-pLL(TFA) in methanol containing 1 M NaOH for 12 h under 35℃. The mixture was then purified by dialysis (membrane MWCO = 6,000-8,000) against pure water. After lyophilization, PEG-pLL was obtained as a white powder. IR780 was then conjugated to PEG-pLL *via* substitution reaction between the meso-chloride and amines^[2]^. IR780 iodide (25 mg), PEG-pLL (50 mg) and TEA (100 μL) were mixed in 8 mL anhydrous DMF. The mixture was kept reacting at 80℃ under Ar atmosphere for 24 h, then precipitated against diethyl ether to collect the PEG-pLL(IR780) polymer. The polymers, including PEG-pLL(TFA), PEG-pLL, and PEG-pLL(IR780), were characterized by ^1^H-NMR in DMSO-*d_6_* and HPLC (JASCXO LC-EXTREMA, JASCO, Japan; column: Superdex 200-10/300GL; mobile phase: 10 mM acetate buffer (pH 3.5); flow rate: 0.75 mL min^-1^; detector: UV 220 nm). The DP of lysine blocks was determined by comparing the integration of the peaks of -CH2-CH2- on PEG (δ = 3.5 ppm) and -CH2-CH2-CH2- on lysine (δ = 1.2 – 2.0 ppm). The number of IR780 conjugated to the polymer was confirmed by comparing the integration of the peaks corresponding to the benzyl groups on IR780 (δ = 6.8-8.6 ppm) and -CH2-CH2- on PEG (δ = 3.5 ppm).

The solubility of the PEG-pLL(IR780) was investigated. The polymer was dispersed in 10 mM HEPES buffer with pH 7.4 or 6.5 at a concentration of 0.1 mg/mL and measured by DLS (Nano-ZS, Malvern, UK) to determine the size distribution.

The absorbance and fluorescence spectra of the polymer were measured. The polymers (0.1 mg/mL) were dissolved in 10 mM HEPES buffer with different pH and scanned by a microplate reader (Spark, Tecan, Switzerland). The fluorescence emission spectra were retrieved with 650 nm excitation light.

*1.4 Computational simulation of polymers*

DFT calculations were performed with Gaussian 16. Geometry optimizations were performed in the solvent (water) with the B3LYP functional^[3]^ and a basis set of 6-311G(d,p), and the IEFPCM method (ε = 78.3553) for water^[4]^.

Molecular dynamics simulations were performed using Gromacs 2020 software to investigate the binding interactions between RNA and polymers. A random RNA sequence with 20 bases (AGCUAGCUAGCUAGCUAGCU) was generated as the representative RNA molecule. Polymers were shortened to 10 pLL units to facilitate the calculation. Amber14sb_OL15 (OL15 & OL3) force field was selected for simulation of nucleic acids. Parameters for the polymers were generated using the online tool AuToFF. The polymers and RNA were placed in a cubic box with a 1 nm distance from the box edges. The system was filled with water molecules (TIP3P). Na+ and Cl- ions were added to the system to neutralize the charges. The simulation was run for 100 ns under 300 K temperature and 1 bar pressure. The trajectory data were analyzed using the trjconv module in Gromacs 2020. The energy, temperature, and pressure variations during the simulation were calculated using the energy module in Gromacs 2020. The RMSD values of each system component were calculated using the rms module. The radius of gyration (Rg) of the RNA was calculated using the gyrate module in Gromacs 2020. Covalent bonds that involved hydrogen atoms were constrained using the LINCS algorithm^[5]^. Atomic contact pairs were defined when any pairs of atoms were within a 3.5 Å cutoff or closer.

*1.5 Preparation and characterization of mRNA-loaded LITS and PIC*

To study the formation of LITS, PEG-pLL(IR780) (1 mg/mL) was dissolved in 10 mM HEPES buffer (pH 4.5) as a stock solution. Luc mRNA (1 mg/mL) was dissolved in 10 mM HEPES buffer (pH 8.5). The polymer solution and mRNA solution were mixed under rigorous vortexing at different ratio between the lysine groups in the polymer and the phosphate groups in mRNA (L/P ratio). The mixture was titrated to pH 7.4 and incubated in 4℃ for 20 min before characterization. The encapsulation of mRNA was confirmed by agarose gel electrophoresis. The samples (20 μg/mL) were added to 1% agarose gel and electrophorized under 100 V × 20 min. The gel was then stained by Midori Green reagent and observed by a transilluminator under blue LED light. The encapsulation efficiency was quantitatively evaluated by Qubit RNA HS assay that detects the unencapsulated mRNAs. LITS sample was added to Qubit RNA working buffer and incubated for 10 min. The fluorescence of the solution was measured using a microplate reader (Spark, Tecan, Switzerland) (Ex/Em: 630/680 nm). The assemble state and size distribution of the samples were evaluated by DLS. The surface charge of the samples was measured by electrophoretic light scattering (Nano-ZS, Malvern, UK). For TEM, samples were diluted in HEPES buffer (pH 7.4) and stained by 2% uranyl acetate, then loaded to 400-mesh copper grids for microscopic observation (JEM-1400, JEOL, Japan).

PIC formed by PEG-pLL and mRNA was prepared as the control formulation. The preparation followed the same protocol as that of LITS, with L/P = 4. PIC were characterized by agarose gel electrophoresis, DLS and electrophoretic light scattering to determine the mRNA encapsulation, size distribution and surface charge, respectively.

The stability of LITS and PIC in different salt concentration was compared by DLS measurement. LITS or PIC samples (10 μg/mL mRNA equivalence) were dispersed into 10 mM HEPES buffer (pH 7.4) containing different concentration of NaCl (0-, 150- and 500-mM) and incubated under 25℃ for 1 h, then loaded for DLS measurement.

*1.6 FCS measurement*

The formation and stability of LITS and PIC in environment containing polyanions were analyzed by FCS. Cy5-labeled Luc mRNA was used to prepare the samples. The samples (10 μg/mL mRNA equivalence) were dispersed into 10 mM HEPES buffers (pH 4.5 or 7.4) with different concentration of dextran sulphate. After incubation for 1 h under 25℃, the samples were loaded into 8-well chambered slides and scanned by FCS mode of a CLSM (LSM880, Zeiss, Germany) with 633 nm excitation. The ACF curves and sample diffusion time values were recorded. The diffusion coefficients of mRNAs in the samples were calculated *via* Einstein-Stokes equitation, as follows:

$$D_{sample}=\frac{D_{Cy5}t_{Cy5}}{t_{sample}}$$

$$d=\frac{k_{B}T}{3\pi\eta D_{sample}}$$

*D_sample_* and *t_sample_* refer to the diffusion coefficient and measured diffusion time of the sample, respectively. *D_Cy5_* and *t_Cy5_* are the diffusion coefficient and measured diffusion time of free Cy5 dye as a standard, in which *D_Cy5_* = 2.8 × 10^-6^ cm^2^ s^-1^ according to reference^[6]^. *d* is the hydrodynamic diameter of the sample. *k_B_* is Boltzmann constant. *T* is the temperature during experiment. $\eta$ is the viscosity of the buffer.

The stability of the samples was also checked in buffer s containing serum. LITS or PIC (10 μg/mL mRNA equivalence) were dispersed into 10 mM HEPES buffers (pH 7.4) containing 10% FBS. The samples were incubated under 25℃ and measured by FCS mode of a CLSM at different time points (0, 20, 40, 60 min).

*1.7 mRNA integrity evaluation*

To test the capability of the formulation to protect mRNA in physiological experiment, RT-PCR of Luc mRNA was performed. Naked mRNA, LITS, and PIC samples (1 μg/mL mRNA equivalence) were dispersed in FBS/D-PBS (*v*/*v* = 1:1) solution and incubated under 37℃ for 30 min. The mRNA in these samples was extracted and purified by RNeasy mini kit, then reverse-transcribed by ReverTraAce qPCR RT Master Mix kit into cDNA. The cDNA samples were analyzed by fluorescence-based quantitative PCR (qPCR) with an ABI Prism 7500 Detector (Applied Biosystems, USA).

To test the effect of light irradiation on the integrity of mRNA loaded in LITS, LITS sample (1 μg/mL mRNA equivalence) was dispersed in 10 mM HEPES (pH 7.4 and 4.5) and irradiated by a 650 nm diode semiconductor laser source (CivilLaser, Naku Technology, China) with a power density at 0.5 W/cm^2^ for determined time points (0, 1.5, 3, and 5 min). The samples were then tested following the same steps above by RT-PCR to evaluate the mRNA integrity.

*1.8 Photosensitizing ability of LITS*

Photothermal conversion and photodynamic property of LITS were evaluated under the irradiation by 650 nm laser (0.5 W/cm^2^). LITS loading Luc mRNA (Luc/LITS) was prepared and dissolved (10 μg/mL mRNA equivalence) in 10 mM HEPES buffer with pH 7.4 or pH 4.5. The temperature of the samples was tracked by a thermal imaging camera (FLIR ONE Pro, FLIR, USA). To evaluate the photodynamic property, DPBF (20 μM) was added into the LITS samples. Upon irradiation with different time, the absorbance of the samples at 430 nm was measured to indicate ROS generation.

*1.9 Safety, cellular uptake, and phototoxicity of LITS*

Safety of PEG-pLL(IR780) and PEG-pLL polymers was firstly evaluated in CT26 cells. CT26 cells were seeded into 96-well plate (10^4^/well) and cultured overnight. The polymers were added into the medium at different concentrations and incubated for another 24 h. The cell viability was then tested by CCK-8 assay.

Cellular uptake of PIC and LITS were evaluated by CLSM. CT26 cells were seeded in 8-well chambered slide (10^4^ cells/well) and cultured overnight. PIC or LITS loading Cy5-labeled Luc mRNA (10 μg/mL mRNA equivalence) was added into the medium. After determined time points, cells were stained by Hoechst 33342 and imaged under CLSM (LSM880, Zeiss, Germany) (Excitation (Ex): 405- and 633-nm/ Emission (Em): 450- and 665-nm respectively). Fluorescence intensity from single cells were measured to indicate the cellular uptake.

Phototoxicity of LITS was firstly tested by calcein/EthD-1 staining assay. Luc/LITS (10 μg/mL mRNA equivalence) was dispersed in cell culture medium and added to CT26 cells seeded in 96-well plate (10^4^ cells/well). After 6 h incubation, cells were washed with D-PBS and irradiated by 650 nm laser (0.5 W/cm^2^) for different time (0, 1 and 5 minutes). The cells were then kept in the medium for another 24 h. Finally, the cells were stained by calcein/EthD-1 staining kit and imaged with fluorescence microscope (BZ-X800, Keyence, Japan) with GFP and TRITC filters. Next, a larger scale screening of the phototoxicity was performed. CT26 cells were incubated with LITS at different concentration for 6 h and irradiated by 650 nm laser (0.5 W/cm^2^) for different time (0-5 min). The cells were then kept in the medium for another 24 h, then analyzed by CCK-8 assay to quantify the cell viability.

*1.10 Intracellular trafficking of LITS and PICs*

The endosomal escape capability of LITS and PICs was evaluated. Samples prepared by Cy5-labeled Luc mRNA (10 μg/mL mRNA equivalence) was added to CT26 cells seeded in 8-well chambered slide (10^4^ cells/well). After 6 h, cells were washed and kept in fresh culture medium. One of the LITS-treated sample was irradiated by 650 nm laser (0.5 W/cm^2^) for 1.5 min. At different time after irradiation (0-, 20-, 60-, 120-, 180-min post irradiation), cells were washed, stained by Hoechst 33342 and Lysotracker Green, then imaged by CLSM (Ex: 405-, 488- and 633 nm/ Em: 450-, 520- and 665-nm, respectively). The colocalization between red and green fluorescence signals was analyzed by Zen software (Zeiss, Germany).

The spatial distribution of LITS after internalization by CT26 cells was evaluated. PEG-pLL(IR780) polymer was labeled with Alexa 405 dye and used to prepare LITS with Cy5-labeled Luc mRNA. CT26 cells were seeded in 8-well chambered slide (10^4^ cells/well) and stained by CellLight Early Endosomes-GFP to label the endosome membrane with green fluorescence. The LITS sample was then dispersed in cell culture medium (10 μg/mL mRNA equivalence) and added into the cells. After 6 h incubation, cells were washed and stained by Lysotracker green, then imaged by the airyscan super-resolution mode of CLSM (Ex: 405-, 488- and 633-nm/ Em: 450-, 520- and 665-nm, respectively).

*1.11 In vitro mRNA transfection efficiency of LITS*

*In vitro* mRNA transfection of LITS was evaluated by EGFP mRNA as a model. EGFP/LITS (2 μg/mL mRNA equivalence) were added to CT26 cells seeded in 48-well plate (5 × 10^4^ cells/well). After 6 h incubation, 650 nm irradiation (0.5 W/cm^2^) with determined duration was applied to the cells. The cells were kept in culture for another 12 h, then analyzed by flow cytometer (FACSAria, BD Biosciences, USA) and imaged by fluorescence microscope to detect the expression of GFP. For ROS inhibition experiment, NAC (2.5 mM) was added to the cell culture medium. To compare the ROS generation level in the cells, Luc/LITS (10 μg/mL mRNA equivalence) were added to CT26 cells seeded in 8-well chambered slide (10^4^ cells/well). After 6 h incubation, cells were stained by H_2_DCFDA and irradiated by 650 nm laser (0.5 W/cm^2^) for defined time (1, 2, 3 and 5 minutes). The cells were then imaged by CLSM (Ex: 488 nm/ Em: 520 nm). Control cells without LITS treatment were subjected to the same irradiation and imaging procedures.

*1.12 mRNA transfection efficiency of LITS via local administration*

BALB/c mice (female, 6 weeks) were subcutaneously (s.c.) inoculated by CT26 cells (10^6^/mouse) at the lower abdomen. Tumor volume was tracked by caliper measurement and calculated by:

$$V= \frac{1}{2}L\times W^{2}$$

*V* is the tumor volume. *L* and *W* are the length and width of the tumor, respectively. At Day 10 post inoculation, the average tumor volume achieved 100 mm^3^. Luc/LITS (2 μg mRNA equivalence) was injected into the tumors. After 6 h, the tumor area was irradiated by 650 nm laser (0.5 W/cm^2^) for 1.5 min. At determined time points post irradiation, luciferin (50 mg/mL dispersed in 200 μL PBS) were intraperitoneally (i.p.) injected to the mice. The mice were then imaged by *in vivo* imaging system (IVIS) (SP-BFM-T1, PerkinElmer, USA) to detect the bioluminescence signal.

*1.13 Pharmacokinetics of LITS upon systemic administration*

BALB/c mice bearing s.c. CT26 tumor (~100 mm^3^) were intravenously (i.v.) injected with LITS loading Cy5-labeled Luc mRNA (10 μg mRNA equivalence). The blood vessels on the earlobe of the mice were observed by IVCLSM (A1R CLSM, Nikon, Japan) (Ex: 633 nm/ Em: 665 nm). For retrieving the quantitative blood circulation profile, ROIs were placed on the vessel area and the fluorescence intensities inside the ROIs were measured continuously. Definite intensity values were normalized by the maximal intensity found in each ROI. After 6 h, the mice were euthanized to harvest the organs and tumors. The tissue samples were imaged by IVIS to detect the fluorescence signals (Ex: 633 nm/ Em: 665 nm).

Blood circulation profiles were also measured by RT-PCR. Mice were treated with the same protocol above but with samples loading non-labeled Luc mRNA. At determined time points, blood samples were collected from orbital vein and immediately proceeded by RNeasy Mini kit to extract the mRNA. The samples were then analyzed by RT-PCR to detect the remaining mRNA.

*1.14 mRNA transfection efficiency of LITS via systemic administration*

Healthy BALB/c mice (female, 6 weeks) were i.v. injected with Luc/LITS (10 μg mRNA equivalence). After 6 h, the liver area was irradiated by 650 nm laser (0.5 W/cm^2^) for 1.5 min. After more 8 h, luciferin (50 mg/mL dispersed in 200 μL PBS) were intraperitoneally (i.p.) injected to the mice. The mice were euthanized after 10 min and the excised organs were imaged by IVIS to detect the bioluminescence signal.

BALB/c mice bearing s.c. CT26 tumor (around 100 mm^3^) were i.v. injected with Luc/LITS (10 μg mRNA equivalence). After 6 h, the tumor area was irradiated by 650 nm laser (0.5 W/cm^2^) for 1.5 min. After more 8 h, luciferin (50 mg/mL dispersed in 200 μL PBS) were intraperitoneally (i.p.) injected to the mice. The mice were euthanized after 10 min and the excised tissues were imaged by IVIS to detect the bioluminescence signal. Another experiment was conducted with EGFP mRNA with the same protocol. The livers and tumors were sampled for cryosection. The samples were embedded and frozen in O.C.T compound (Sakura Finetek, USA) and sliced to 10 μm thickness by a cryostat (CM3050, Leica, Germany). The slices were mounted in antifade mountant with DAPI (Thermal Fisher, USA) and imaged by CLSM (Ex: 405- and 488-nm/ Emi: 450- and 520-nm, respectively).

*1.15 Performance of IL-2/LITS in CT26 tumor model*

The *in vitro* transfection efficiency of IL-2/LITS was evaluated in CT26 cells. LITS (1 μg/mL mRNA equivalence) was added into CT26 cells seeded in 96-well plate (10^4^ cells/well) and incubated for 6 h. The cells were then irradiated by 650 nm laser (0.5 W/cm^2^) for different duration. After 8 h, the culture medium supernatants were collected, and the IL-2 concentration was measured by ELISA kit.

BALB/c mice bearing s.c. CT26 tumor (around 100 mm^3^) were i.v. injected with IL-2/LITS (10 μg mRNA equivalence). After 6 h, the tumor area was irradiated by 650 nm laser (0.5 W/cm^2^) for 1.5 min. After more 8 h, the mice were euthanized to collect the blood, liver and tumor samples. Blood was sampled from the abdominal artery and kept in heparinized tubes, then centrifuged (400g × 5 min) to separate the plasma. The liver and tumor samples were homogenized in D-PBS supplemented with protease inhibitor. The homogenized tissue suspensions were centrifuged (10000 g × 10 min) to collect the supernatants. The plasma and tissue lysate samples were measured by ELISA kit to quantify the IL-2 concentration.

Immune analysis and antitumor efficacy test were conducted with a multiple administration schedule. BALB/c mice (female, 6 weeks) were subcutaneously (s.c.) inoculated by CT26 cells (10^6^/mouse) at the lower abdomen on Day 0. Treatments were conducted on Days 9, 11, and 13. For each treatment, mice were i.v. injected with IL-2/LITS (10 μg mRNA equivalence). After 6 h, the tumor area was irradiated by 650 nm laser (0.5 W/cm^2^) for 1.5 min. The tumor volume was tracked during the experiment. On Day 17, mice were euthanized to collect blood, liver, spleen, and tumor samples. Blood samples were centrifuged (400g × 5 min) to separate the plasma and the blood cells. The plasma was measured by ELISA and blood analyzer (DRI-CHEM NX10N, Fujifilm, Japan) to quantify the cytokine concentrations and toxicity markers. The blood cells were suspended in blood cell lysis buffer to remove the blood cells before staining for flow cytometry. The tumors were cut to two parts for separated measurements by ELISA and flow cytometry. For ELISA, the tumors were homogenized and centrifuged (10,000 g × 10 min) to collect the supernatants. For flow cytometry, tumors were digested in RPMI medium containing 0.2% collagenase to prepare cell suspension. Livers were also digested in RPMI medium containing 0.2% collagenase to prepare cell suspension. The lymphocytes in the liver cell samples were isolated by gradient centrifuge^[7]^. Spleens samples were homogenized on a 100 μm cell strainer and treated by red blood cell lysis buffer. For flow cytometry analysis, the single cell suspensions were blocked by anti-mouse CD16/32 and stained by Live/Dead fixable near-IR dead cell staining dye, PE-Cy7-anti-mouse CD45, PE-anti-mouse CD3, FITC-anti-mouse CD4, and PerCP-eFluor 710-anti-mouse CD8. The samples were then treated by Foxp3/transcription factor staining buffer for permeabilization and stained by Alexa 647-anti Foxp3. The cells were loaded to flow cytometer (FACSAria, BD Biosciences, USA) and analyzed *via* the gating strategy shown in **Supplementary Figure S38-41**. T cell sub-populations have been analyzed by Uniform Manifold Approximation and Projection (UMAP) plugin of FlowJo software (BD Biosciences, USA) for dimension reduction analysis. The tumors were also evaluated by histological analysis. The samples were embedded and frozen in O.C.T compound and sliced to 10 μm thickness by cryostat. The slices were stained by H&E staining kit or Alexa 647-anti-mouse CD8α, then observed by fluorescence microscope (empty filter and Cy5 filter for H&E-stained samples and antibody-stained samples, respectively).

*1.16 Performance of IL-2/LITS in dual primary CT26 tumor model*

BALB/c mice (female, 6 weeks) were subcutaneously (s.c.) inoculated by CT26 cells (10^6^/injection) at both sides of the lower abdomen on Day 0. Treatments were conducted on Days 7, 9, and 11. For each treatment, mice were i.v. injected with IL-2/LITS or Luc/LITS (10 μg mRNA equivalence). After 6 h, only one of the tumors was irradiated by 650 nm laser (0.5 W/cm^2^) for 1.5 min. The tumor volume was tracked during the experiment. On Day 17, mice were euthanized to collect the tumors for ELISA and flow cytometry test with the same protocol in last section.

*1.17 Performance of IL-2/LITS in primary CT26 tumor model with prolonged irradiation*

*In vitro* experiment was firstly conducted in CT26 cells. Luc/LITS (1 μg/mL mRNA equivalence) was added into CT26 cells seeded in 8-well chambered slid (10^4^ cells/well) and incubated for 6 h. The cells were then irradiated by 650 nm laser (1.0 W/cm^2^) for different duration (0, 1.5, 3.0 and 5.0 minutes). After 12 h, the cells were stained by Hoechst 33342 and Alexa 488-anti-calreticulin, then imaged by CLSM (Ex: 405- and 633-nm/ Em: 450- and 665 nm, respectively).

*In vivo* experiments were conducted in BALB/c mice bearing s.c. CT26 tumor (around 100 mm^3^). To confirm ICD, mice were i.v. injected with Luc/LITS (10 μg mRNA equivalence). After 6 h, the tumors were irradiated by 650 nm laser (0.5 W/cm^2^) for different durations (0, 1.5 and 3.0 minutes). The temperature of the tumor was tracked by thermal imaging camera. After 24 h, the tumors were collected for cryosections. The sliced samples were stained by Alexa 488-anti-calreticulin and TUNEL assay, then observed by fluorescence microscope. To evaluate the correlation between IL-2 expression level and light irradiation time, mice were i.v. injected Luc/LITS (10 μg mRNA equivalence). After 6 h, the tumors were irradiated by 650 nm laser (0.5 W/cm^2^) for different durations (0, 1.5, 3.0 and 5.0 minutes). After more 8 h, tumor samples were homogenized to prepare the lysate supernatants for ELISA measurement quantifying the IL-2 concentration.

The antitumor efficacy of treatment with different irradiation time was evaluated. BALB/c mice (female, 6 weeks) were subcutaneously (s.c.) inoculated by CT26 cells (10^6^ cells/mouse) at the lower abdomen on Day 0. Treatments were conducted on Day 7, 9, and 11. For each treatment, mice were i.v. injected with Luc/LITS or IL-2/LITS (10 μg mRNA equivalence). After 6 h, the tumor area was irradiated by 650 nm laser (0.5 W/cm^2^) for different time (0, 1.5 and 3.0 minutes). The tumor volume was tracked during the experiment. On Day 20, some tumor samples were collected for histological analysis. The tumors were sliced to 10 μm cryosection and stained by H&E staining kit, TUNEL assay kit, and Alexa 647-anti-mouse CD8α + FITC-anti-mouse/human Granzyme B and observed by fluorescence microscope. Mice with tumor exceeding 1000 mm^3^ were euthanized and counted as death to record the survival curves.

*1.18 Performance of IL-2/LITS in metastatic 4T1 tumor model*

Metastatic 4T1 tumor model was established with previously reported method^37^. Briefly, an orthotopic 4T1 primary tumor model was generated in BALB/c mice (female, 6 weeks) by inoculating 10^6^ cells into the mammary fat pad on Day 0. On Day 15, the primary tumors were surgically removed. Treatments were administered on Days 18, 20, and 22. For each treatment, mice received an i.v. injection of Luc/LITS or IL-2/LITS (10 μg mRNA equivalence). Six-hours post-injection, the chest front and back areas were irradiated with a 650 nm laser (0.5 W/cm^2^) for 3 min each. At 8 h after the first treatment, 3 mice from each group were euthanized to collect lungs for ELISA to quantify the IL-2 levels. On Day 30, some mice from each group were euthanized to collect lung samples for observation and histological evaluation. The lungs were sliced to 10 μm cryosection and stained by H&E staining kit. The remaining mice were monitored for tracking the survival.

1.19 Statistical analysis

Statistical analyses were performed by Prism software (V10.2.0). Details of the analysis are specified in the context describing the experiment results accordingly.

1. **Supplementary results**


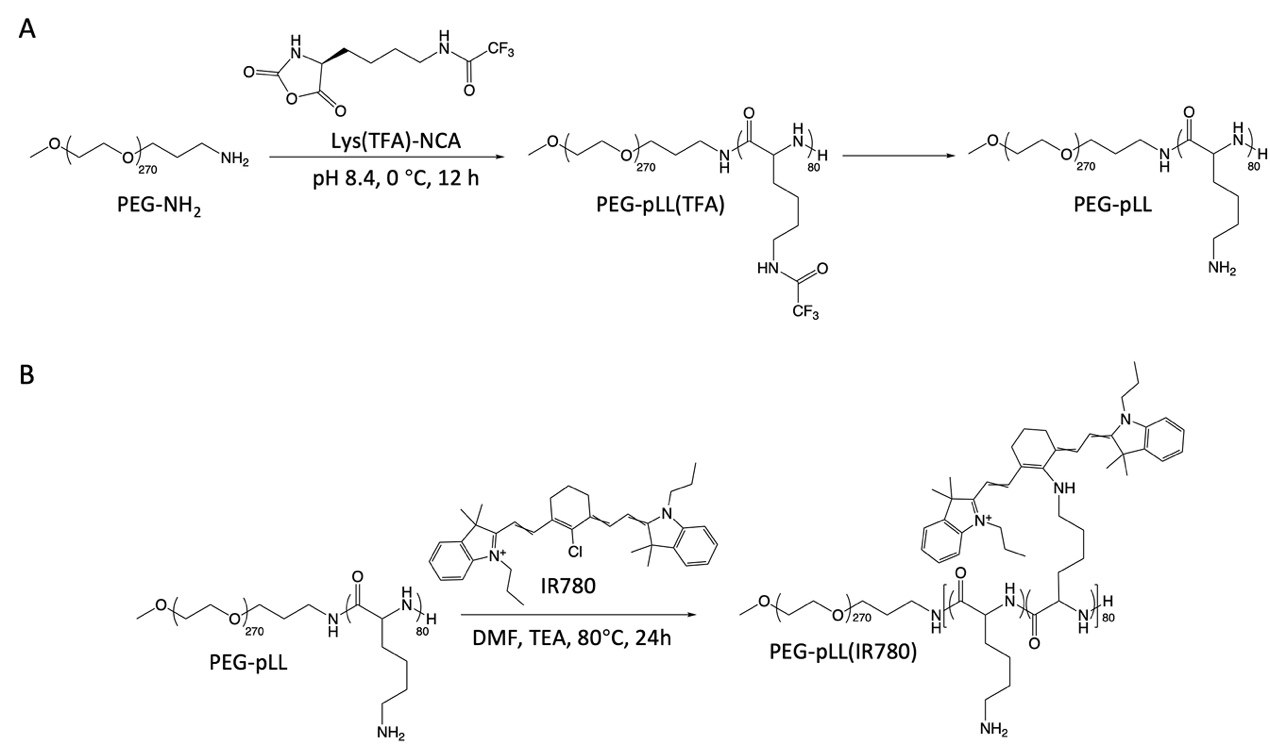


**Scheme S1.** Synthesis route of PEG-pLL(IR780). **A.** PEG-pLL was synthesized *via* ROP of Lys(TFA)-NCA initiated by PEG-NH_2_. **B.** IR780 was conjugated to PEG-pLL *via* the substitution reaction between the meso-chloride and the amine.


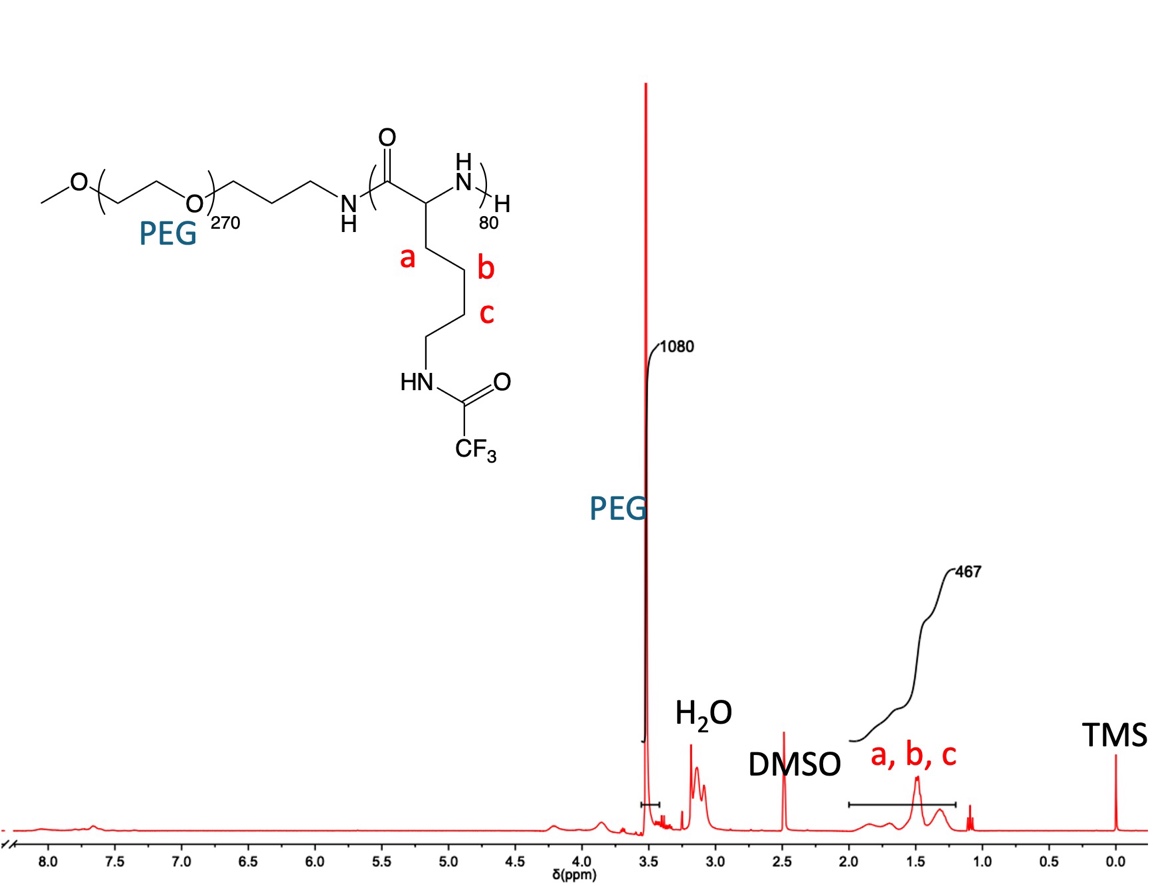


**Figure S1.** ^1^H-NMR results of PEG-pLL(TFA). The DP of lysine blocks was determined by comparing the integration of the peaks of -CH2-CH2- on PEG (δ = 3.5 ppm) and -CH2-CH2-CH2- on lysine (δ = 1.2 – 2.0 ppm, peak a, b, and c) and calculated to be ~80.


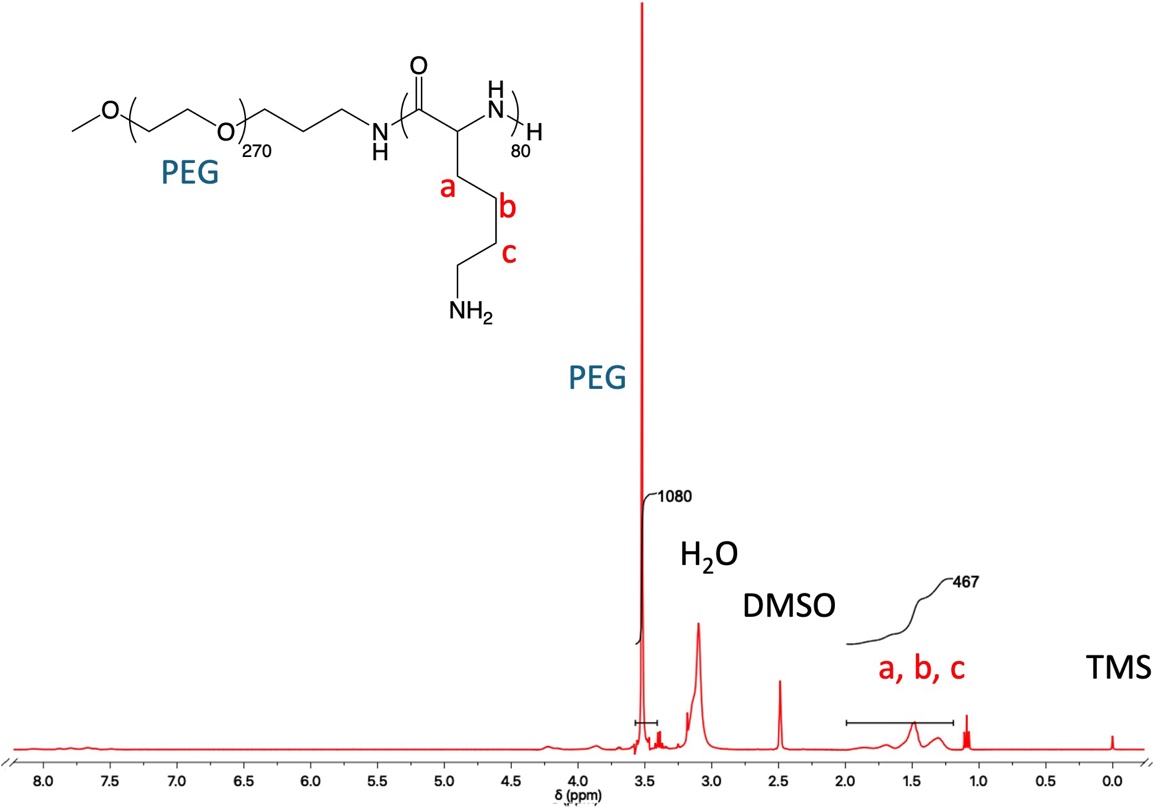


**Figure S2.** ^1^H-NMR results of deprotected PEG-pLL. The DP of lysine blocks was determined by comparing the integration of the peaks of -CH2-CH2- on PEG (δ = 3.5 ppm) and -CH2-CH2-CH2- on lysine (δ = 1.2 – 2.0 ppm, peak a, b, and c) and calculated to be ~80.


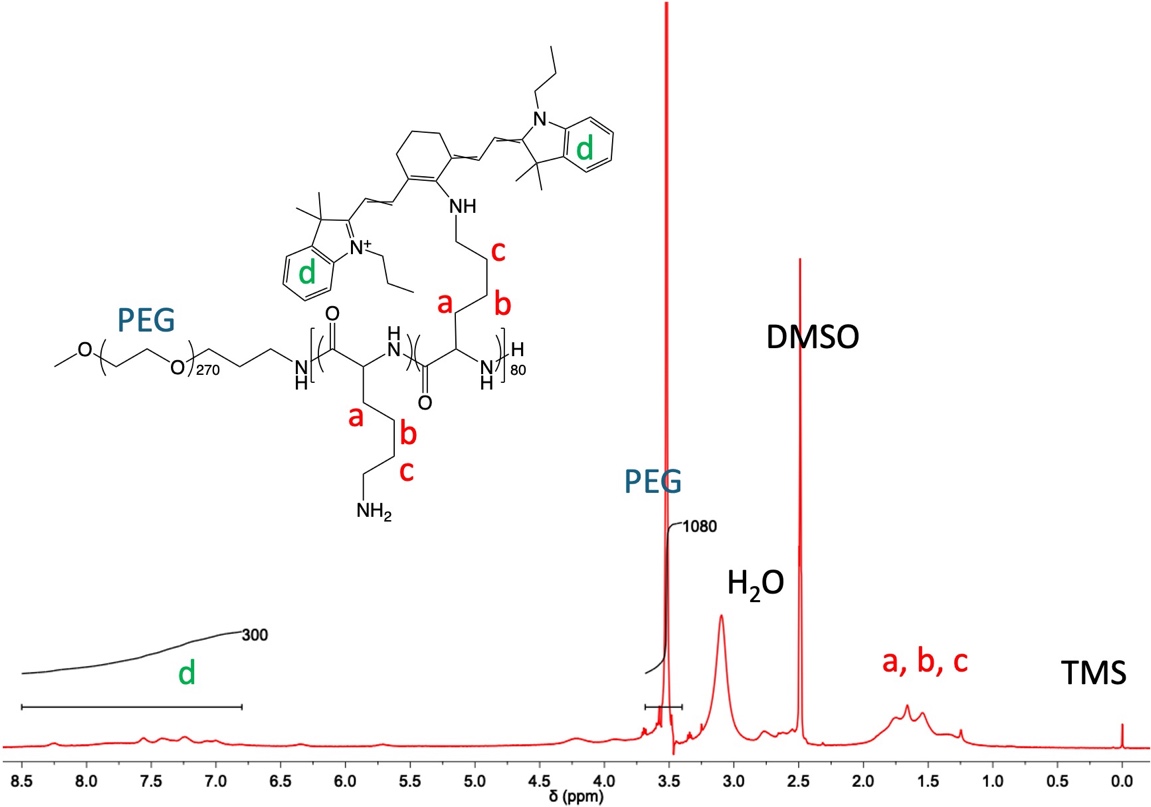


**Figure S3.** ^1^H-NMR results of PEG-pLL(IR780). The conjugation of IR780 to the polymer was confirmed by the appearance of the peaks corresponding to the benzyl groups on IR780 (δ = 6.8-8.6 ppm, peak d). The number of IR780 groups conjugated to one polymer molecule was determined to be 38.

**Figure S4.** SEC analysis results of PEG-pLL and PEG-pLL(IR780).


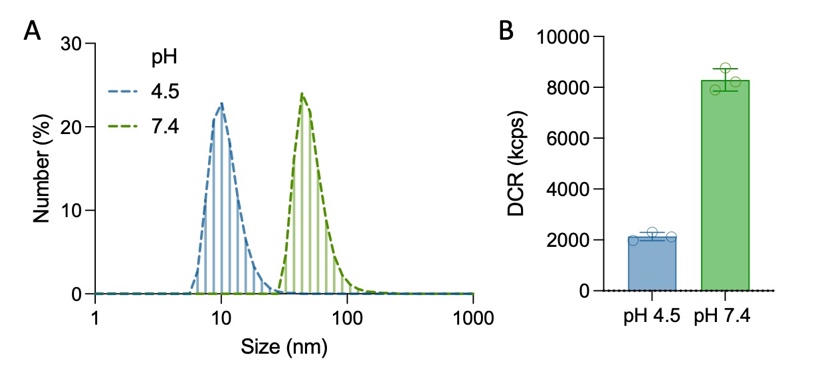


**Figure S5.** Representative DLS measurement results of the PEG-pLL(IR780) polymers dissolving in buffers with different pH. **A.** Representative size distribution of the polymer solutions. **B.** Derived count rate (DCR) values showing the light scattering intensity of the samples. Data are plotted as the mean ± S.D., *n* = 3 independent samples.

**Figure S6.** Confirmation of mRNA encapsulation. **A.** Representative agarose gel electrophoresis results of LITS (upper panel) and PIC (lower panel) samples prepared with different L/P ratio (1 to 6). The red arrow indicates the band of free mRNA. B. Quantitative encapsulation efficiency of the samples (L/P ratio = 4) evaluated by Qubit RNA HS assay. Data are plotted as the mean ± S.D., *n* = 3 independent samples.


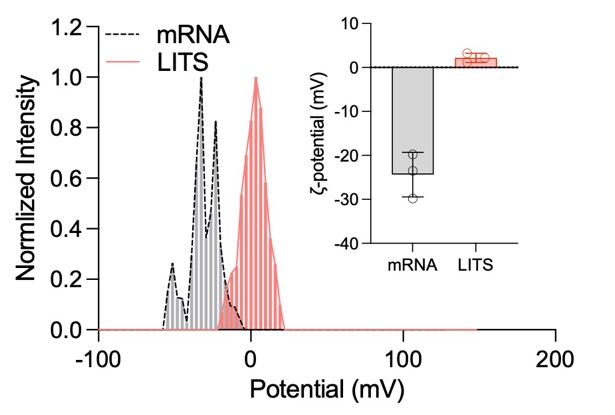


**Figure S7.** ζ-potential distribution and average value of free mRNA and LITS. Data are plotted as the mean ± S.D., *n* = 3 independent samples.

| **Table S1.** Summary of FCS measurement results | | | |
| --- | --- | --- | --- |
| Sample | Diffusion time (μs)^a^ | Diffusion coefficient (μm^2^/s) | Count per molecule (kHz) |
| Cy5 | 98.7 ± 2.9 | 280^b^ | 0.8 ± 0.2 |
| Luc mRNA | 1355.3 ± 87.3 | 20.4 ± 1.4 | 2.9 ± 0.1 |
| LITS | 2501.0 ± 56.3 | 11.1 ± 0.3 | 2.7 ± 0.1 |
| PIC | 2990.3 ± 234.5 | 9.3 ± 0.7 | 3.3 ± 0.4 |
| ^a^ Data are shown as the mean ± S.D., *n* = 3 measurements.  ^b^ Reported result from reference^[8]^. | | | |


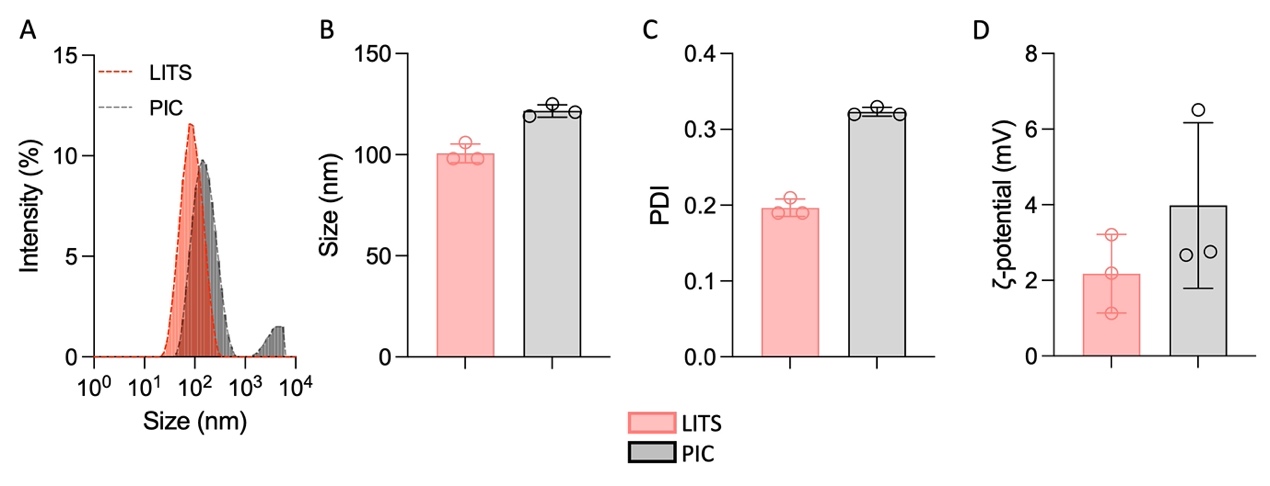


**Figure S8.** Characterization of LITS and PIC. **A.** Representative size distribution measured by DLS. **B.** Average size, **C.** PDI, and **D.** ζ-potential of the samples. Data are plotted as the mean ± S.D., *n* = 3 independent samples.


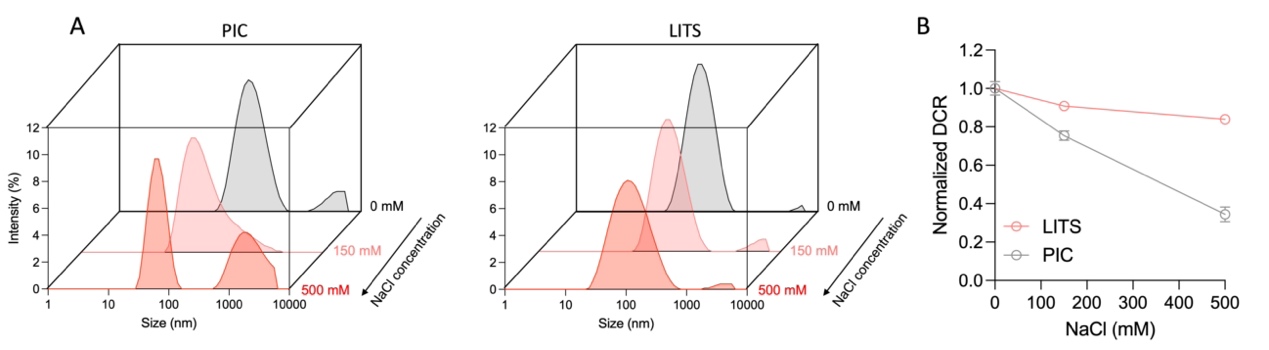


**Figure S9.** Stability of LITS and PIC in the presence of electrolyte. **A.** Representative size distribution profiles of mRNA-loaded samples under different NaCl concentration. **B.** The light scattering intensity (DCR) of the samples. Data are plotted as the mean ± S.D., *n* = 3 independent measurements.

**Figure S10.** Stability of LITS and PIC in buffer containing serum. The samples were diluted in HEPES buffer (10 mM, pH 7.4) containing 10% FBS and incubated under 25℃. Diffusion coefficients of the particles were measured by FCS. Data are plotted as the mean ± S.D., *n* = 3 independent measurements.


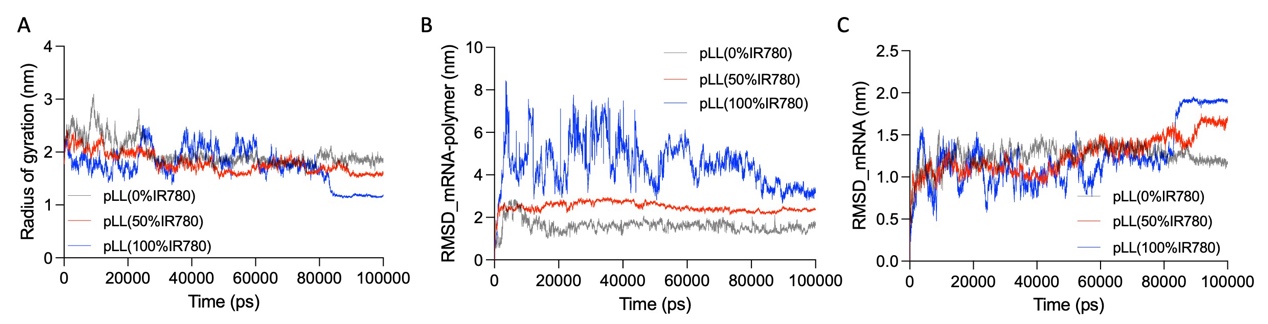


**Figure S11.** Quantitative analysis results from the molecular dynamic simulations showing **A**) the radius of gyration of mRNA, **B**) RMSD of the mRNA-polymer system during the simulation time, and **C**) RMSD of the mRNA molecule.


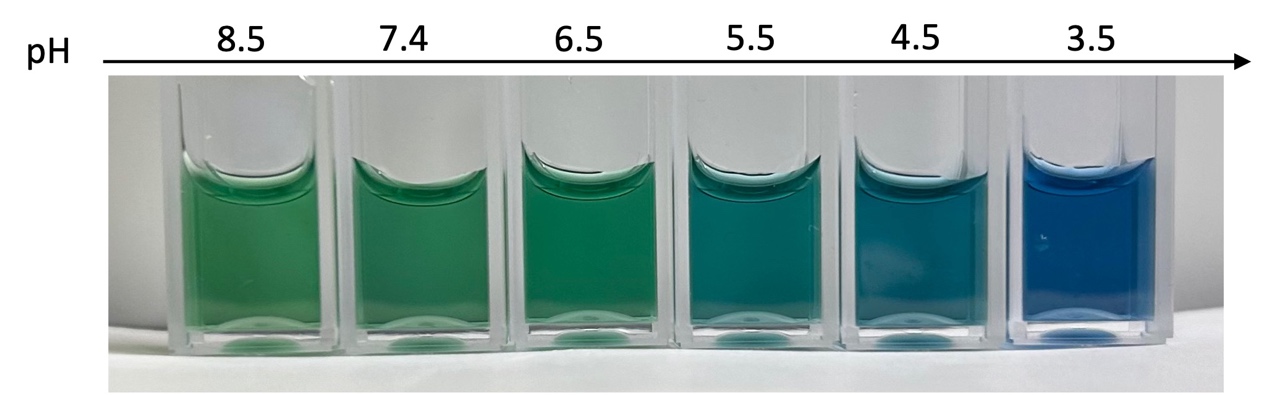


**Figure S12.** Color change of the PEG-pLL(IR780) polymer dissolving in buffer with different pH.


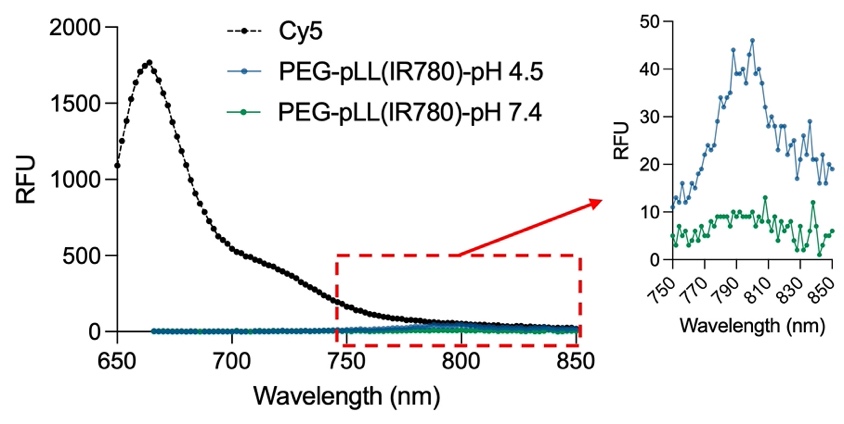


**Figure S13.** Representative fluorescence emission spectra of PEG-pLL(IR780) (0.1 mg/mL) dispersed in pH 4.5 and pH 7.4 HEPES buffer (excitation wavelength = 650 nm). Cy5 sample at the equivalent concentration with IR780 was measured as a standard.


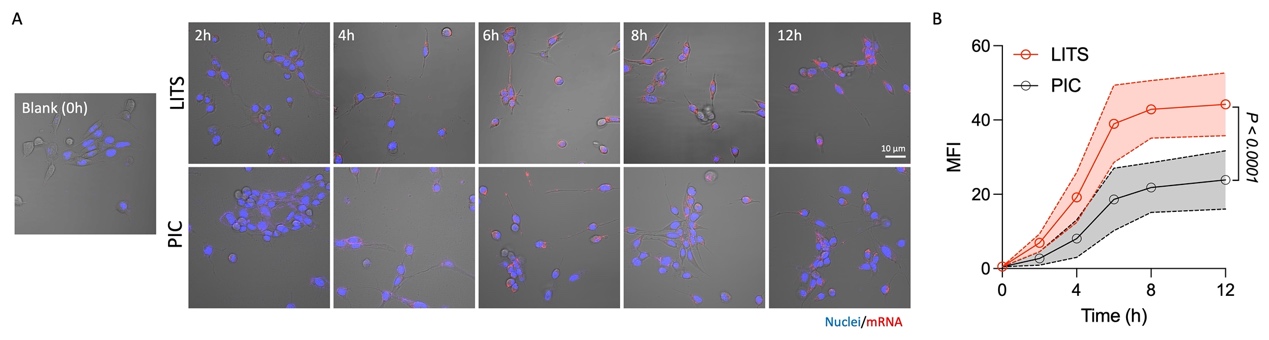


**Figure S14.** Cellular uptake of PIC and LITS by CT26 cells. **A.** Representative microscopic images of the cells after different incubation time with PIC or LITS. **B.** Mean fluorescence intensity (MFI) in the cells upon different incubation time. Data are plotted as the mean ± S.D., *n* = 10 independent images analyzed. The values at 12 h were compared *via* Student’s *t*-test.

**Figure S15.** Cytotoxicity of PEG-pLL(IR780) and PEG-pLL in CT26 cells measured by CCK method. Data are plotted as the mean ± S.D., *n* = 4 independent samples.


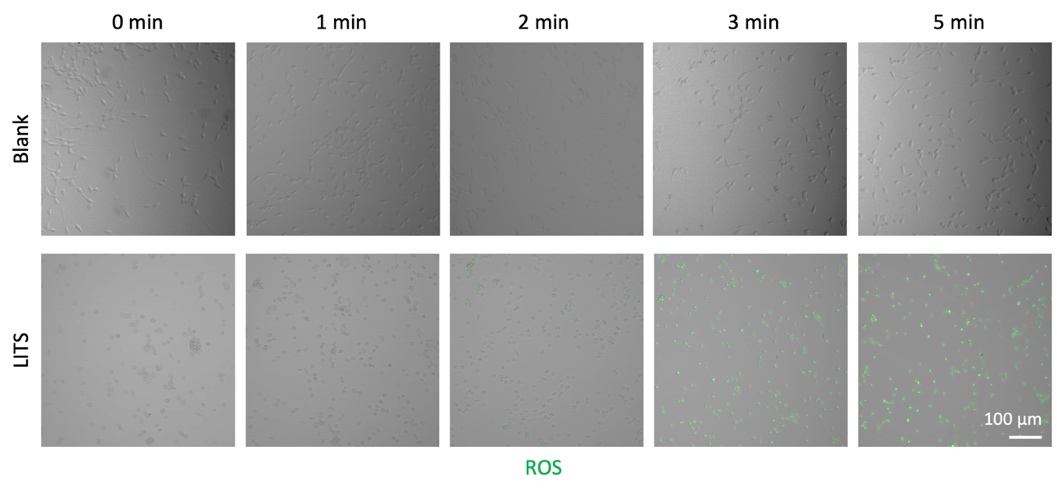


**Figure S16.** Representative fluorescence microscopic images showing the ROS generation in non-treated or LITS-treated CT26 cells after different irradiation time. The ROS level was visualized by dichlorodihydrofluorescein diacetate (H_2_DCFDA) probe.


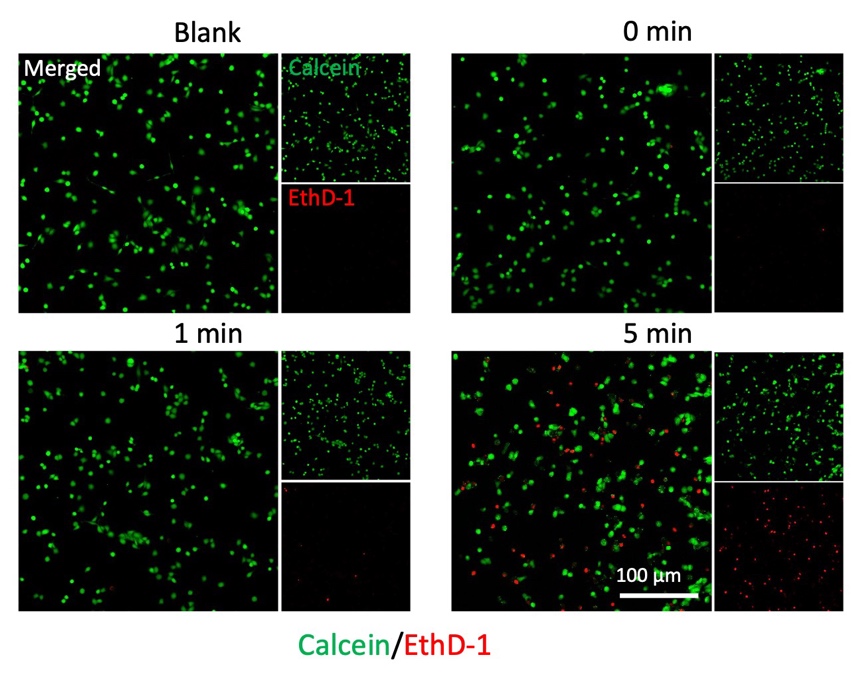


**Figure S17.** Representative microscopic images of the calcein/EthD-1 staining indicating the viability of LITS-treated cells upon irradiation by 650 nm laser (0.5 W/cm^2^) with different time.


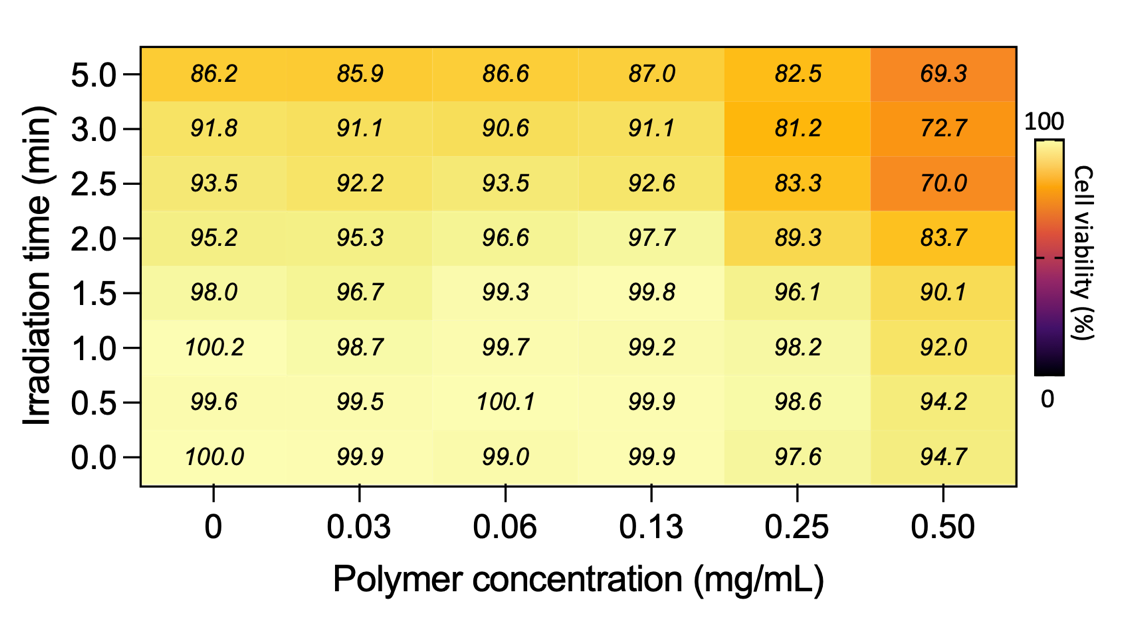


**Figure S18.** Cell viability screening under different irradiation time and polymer concentration. Data are shown as the mean, *n* = 3 independent samples.


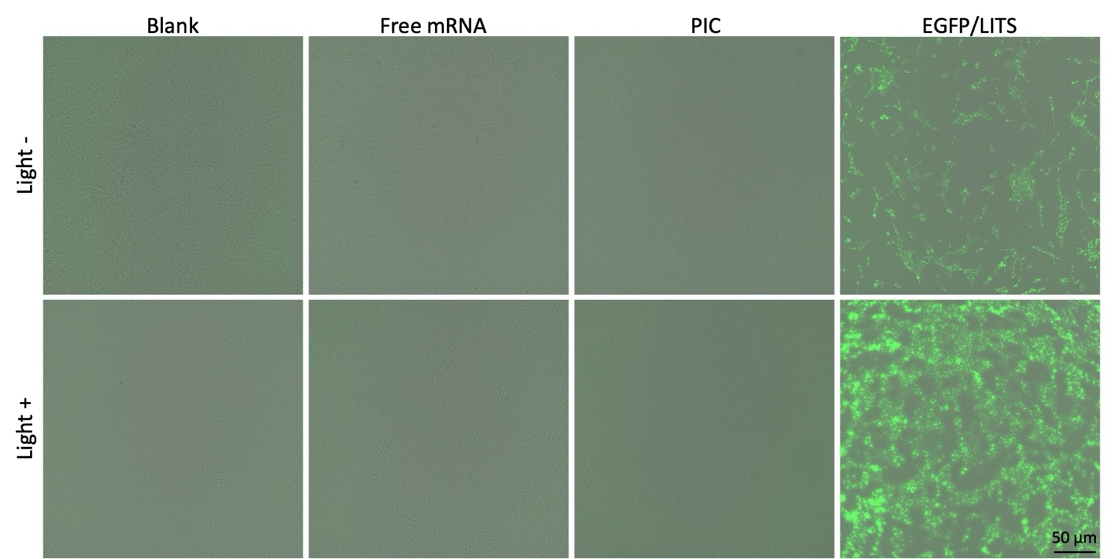


**Figure S19.** Representative fluorescence microscopic images showing the EGFP expression in CT26 cells after treating with different samples. Laser irradiation was applied for 1.5 min.


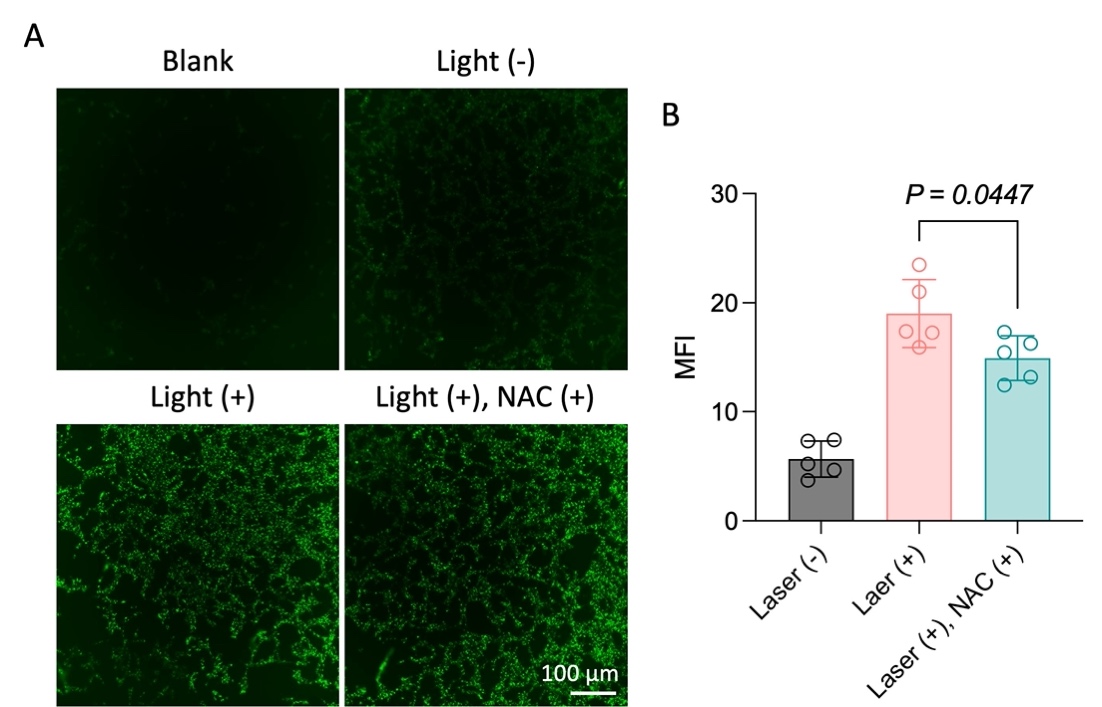


**Figure S20.** The effect of ROS inhibitor *N*-acetyl-*L*-cysteine (NAC) on the performance of EGFP/LITS. **A.** Representative fluorescence microscopic images showing the EGFP expression in CT26 cells after different treatment. **B.** Mean fluorescence intensity of the images. Data are plotted as the mean ± S.D., *n* = 5 independent images analyzed. The results were compared *via* one-way ANOVA.

**Figure S21.** Bioluminescence intensities from the tissue samples presented in **Figure 4H**. Data are plotted as the mean ± S.D., *n* = 3 independent animals. The results were compared *via* Student’s *t*-test.

**Figure S22.** Representative fluorescence microscopic images of tissue sections after treatment with EGFP/LITS. Light irradiation was applied to the liver area at 6 h post injection.

**Figure S23.** *In vitro* expression of IL-2 from CT26 cells after treatment with IL-2/LITS. Data are plotted as the mean ± S.D., *n* = 3 independent samples.

**Figure S24.** Cytokine levels in the tumors from the experiment of **Figure 5C**. Data are plotted as the mean ± S.D., *n* = 5 independent animals. The results were compared *via* one-way ANOVA.


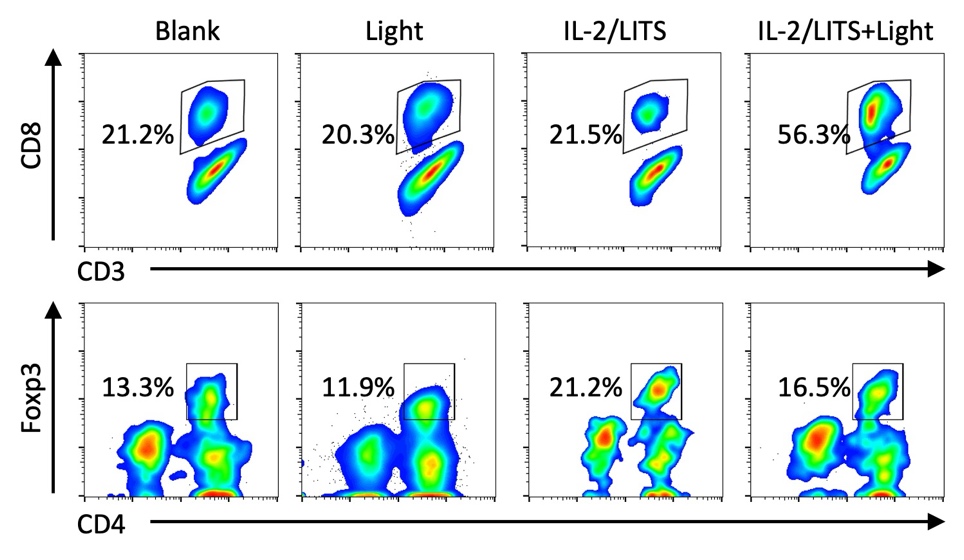


**Figure S25.**Representative flow cytometry plots showing the population fractions of CD8+ cytotoxic T cells and CD4+Foxp3+ Tregs in the tumors from the experiment of **Figure 5C**.


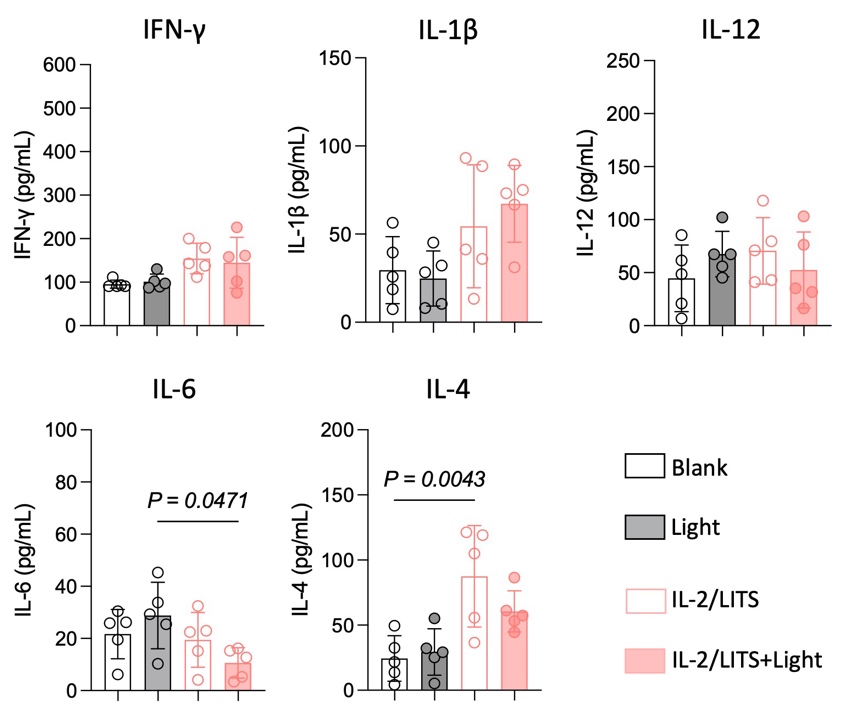


**Figure S26.** Cytokine concentrations in the blood samples from the experiment of **Figure 5C**. Data are plotted as the mean ± S.D., *n* = 5 independent animals. The results were compared *via* one-way ANOVA.


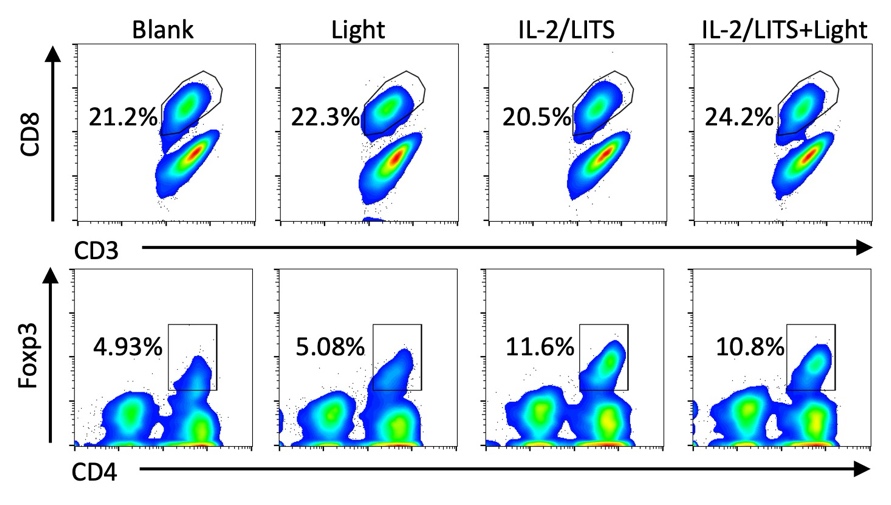


**Figure S27.** Representative flow cytometry plots showing the population fractions of CD8+ cytotoxic T cells and CD4+Foxp3+ Tregs in the blood samples from the experiment of **Figure 5C**.


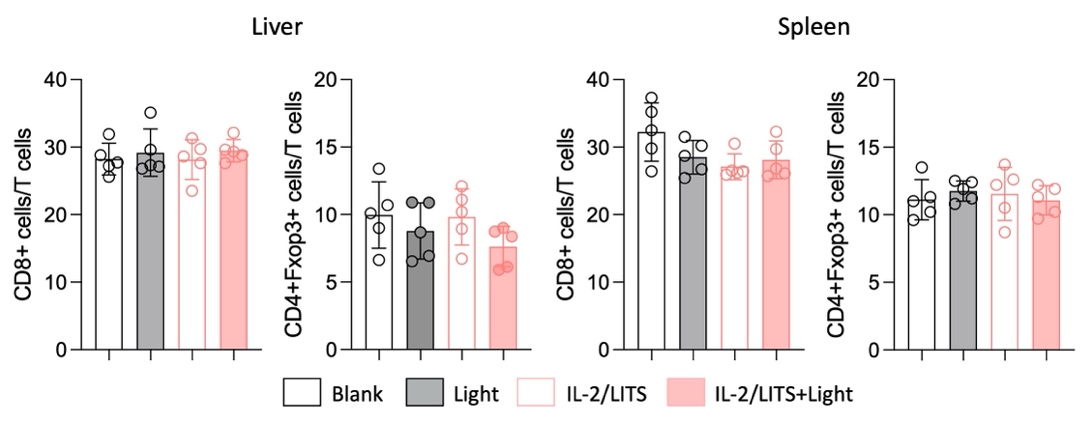


**Figure S28.** Population fractions of CD8+ T cells and CD4+Foxp3+ Tregs in the liver and spleen samples from the experiment in **Figure 5C**. Data are plotted as the mean ± S.D., *n* = 5 independent animals. The results were compared *via* one-way ANOVA and no significant difference could be found among any groups.


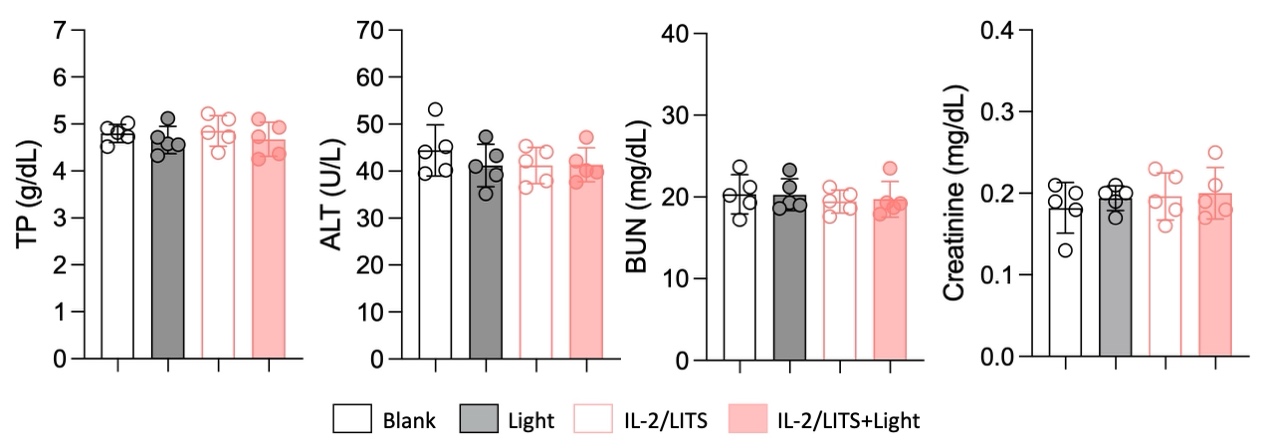


**Figure S29.** Toxicity markers level in the blood samples from the experiment in **Figure 5C**. Data are plotted as the mean ± S.D., *n* = 5 independent animals. The results were compared *via* one-way ANOVA and no significant difference could be found among any groups.


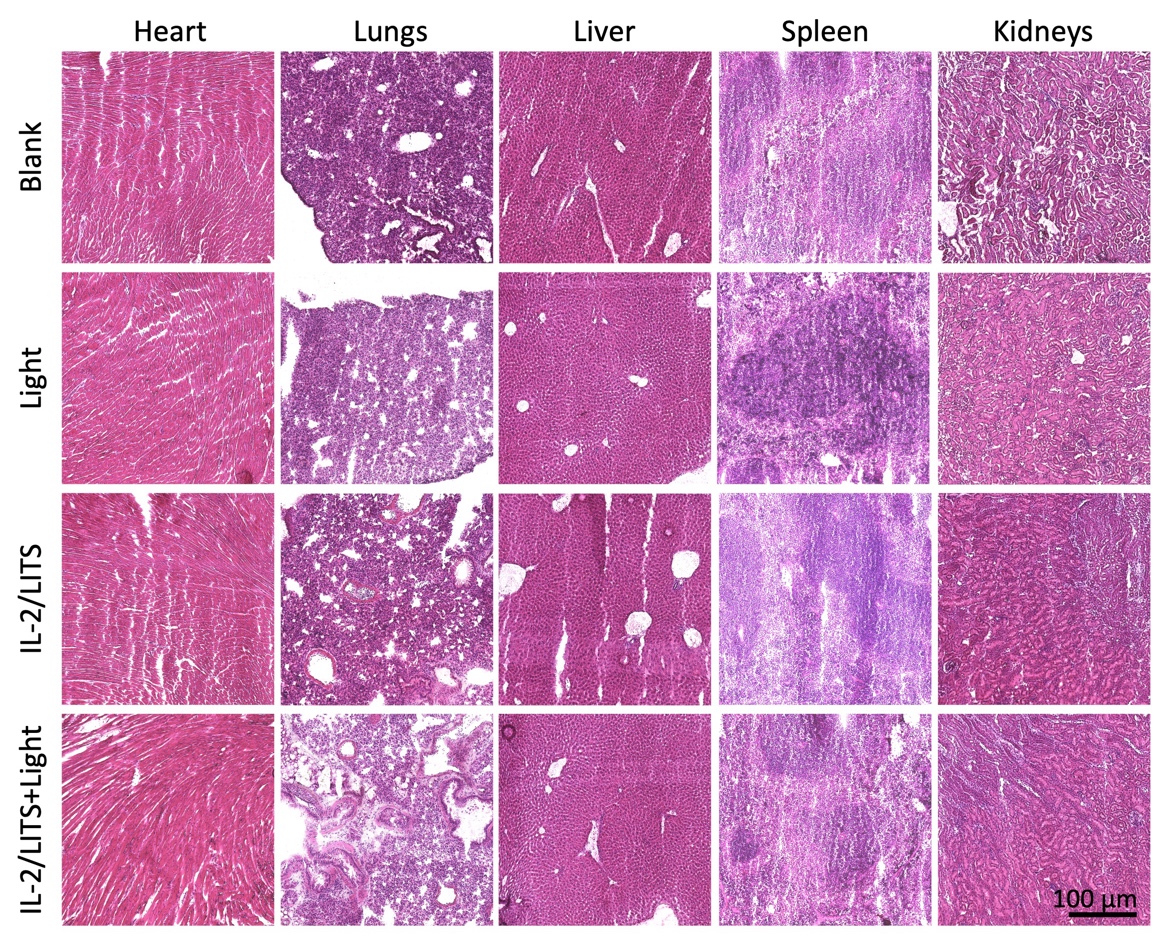


**Figure S30.** Representative H&E histological analysis of the organ samples collected from the experiment in **Figure 5C**.

**Figure S31.** Cytokine levels in the tumors from the experiment in **Figure 6D**. Data are plotted as the mean ± S.D., *n* = 6 independent animals. The results were compared *via* one-way ANOVA.


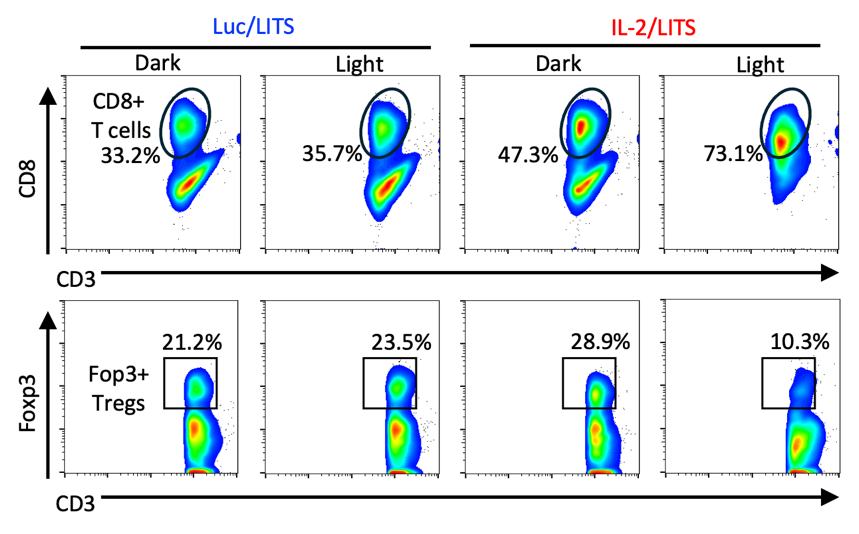


**Figure S32.** Representative flow cytometry plots showing the fractions of CD8+ cytotoxic T cells and Foxp3+ Tregs in the tumors from the experiment in **Figure 6D**.


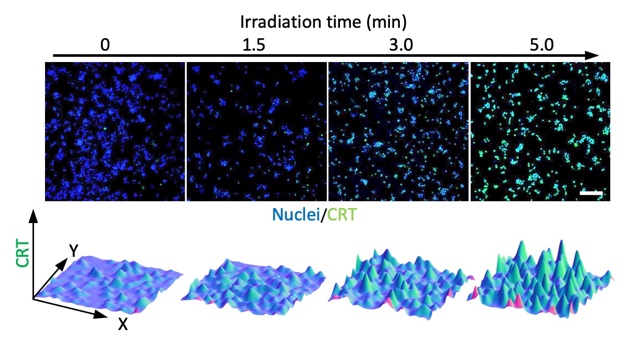


**Figure S33.** Representative confocal microscopic images showing the expression of calreticulin (CRT) on CT26 cells upon different irradiation time after Luc/LITS treatment.


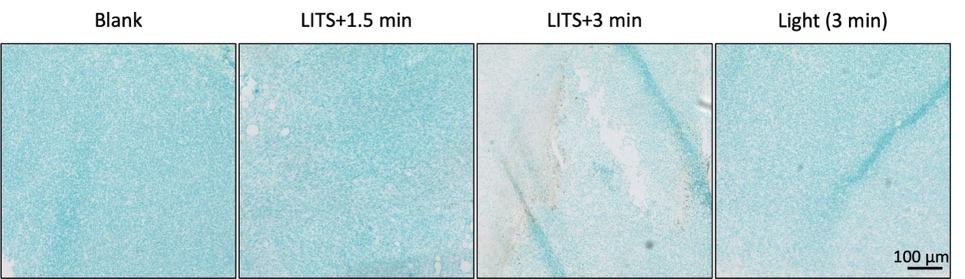


**Figure S34.** Representative TUNEL assay staining of Luc/LITS-treated tumors after different irradiation time.


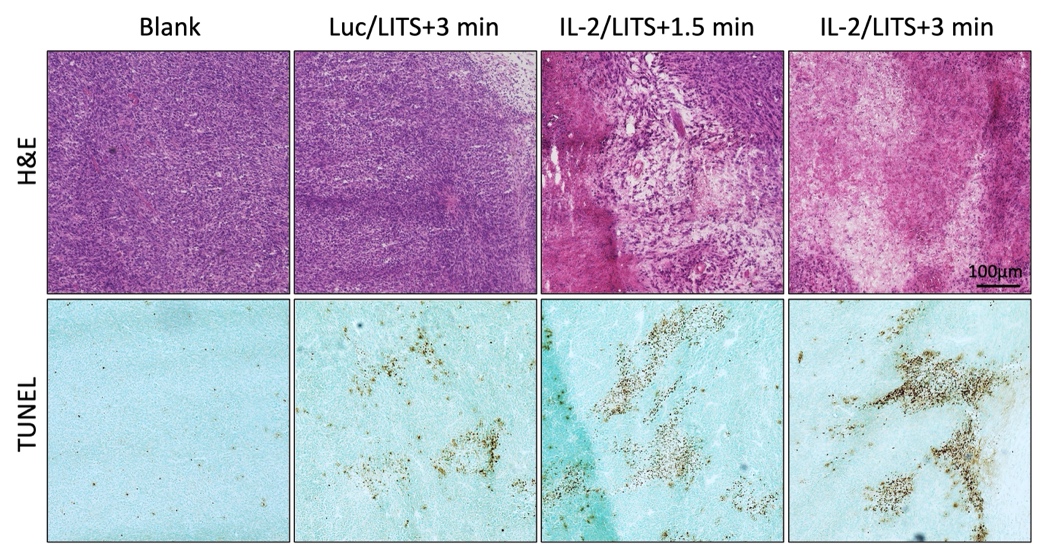


**Figure S35.** Representative H&E and TUNEL assay staining of tumor samples on Day 20 in **Figure 7E**.

**Figure S36.** Bodyweight change of the animals during the experiment of **Figure 7E**. Data are plotted as the mean ± S.D., *n* = 5 independent animals.

**Figure S37.** Bodyweight change of the animals during the experiment of **Figure 7I**. Data are plotted as the mean ± S.D., *n* = 6 independent animals.


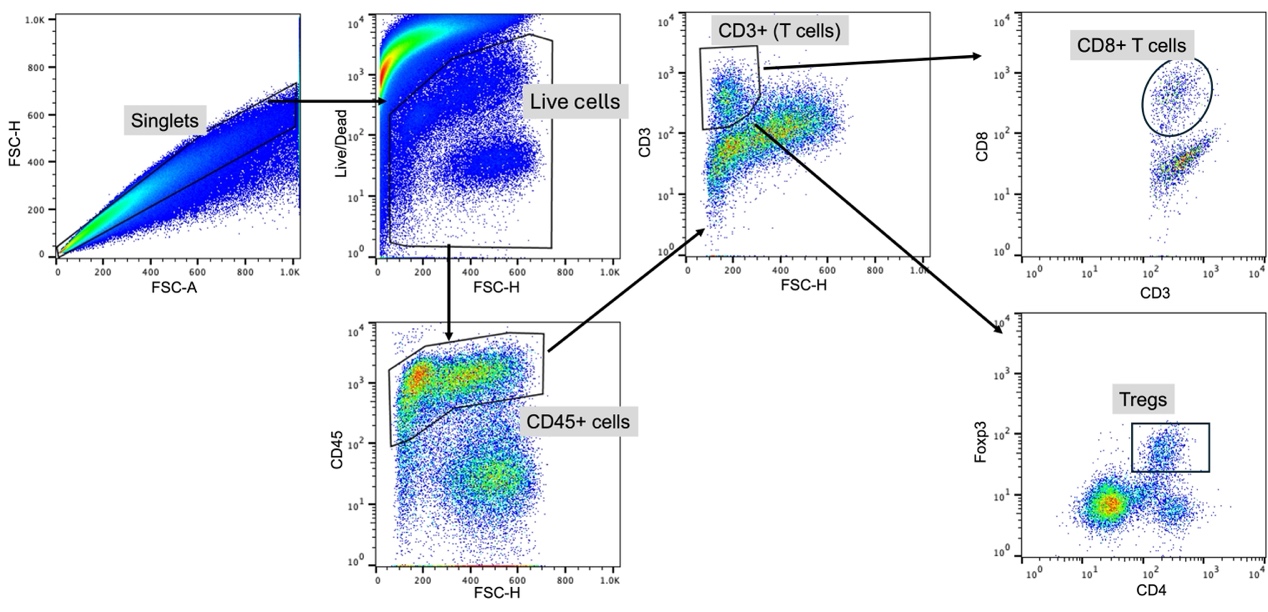


**Figure S38.** Gating strategy for the analysis of tumor-infiltrating lymphocytes.


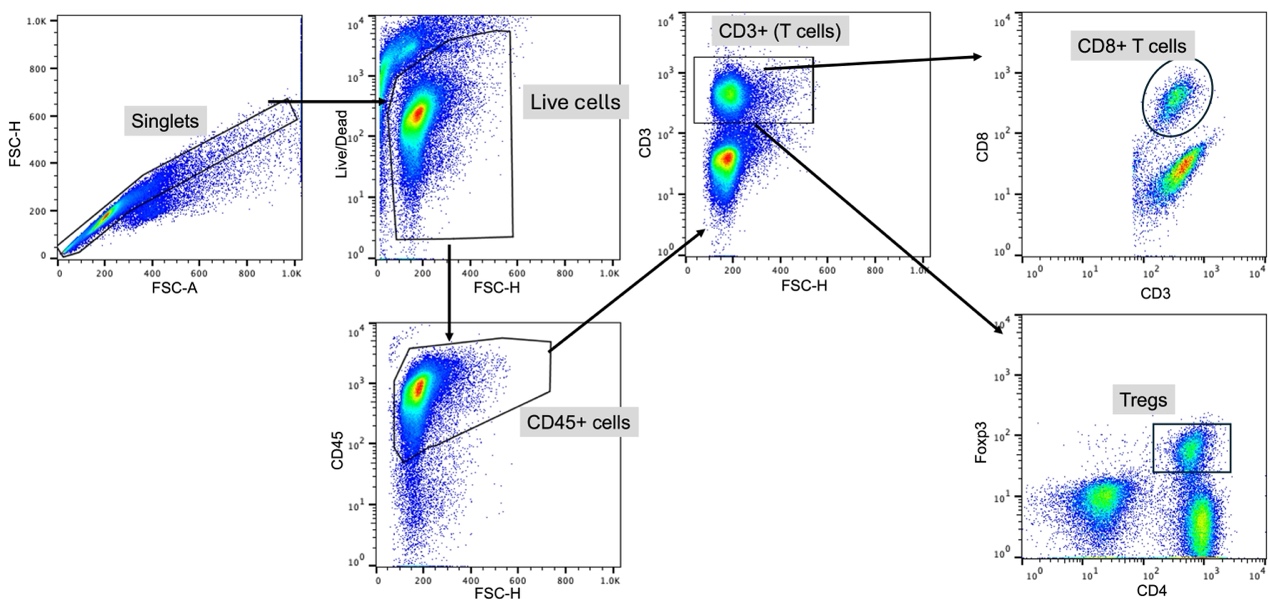


**Figure S39.** Gating strategy for the analysis of splenocytes.


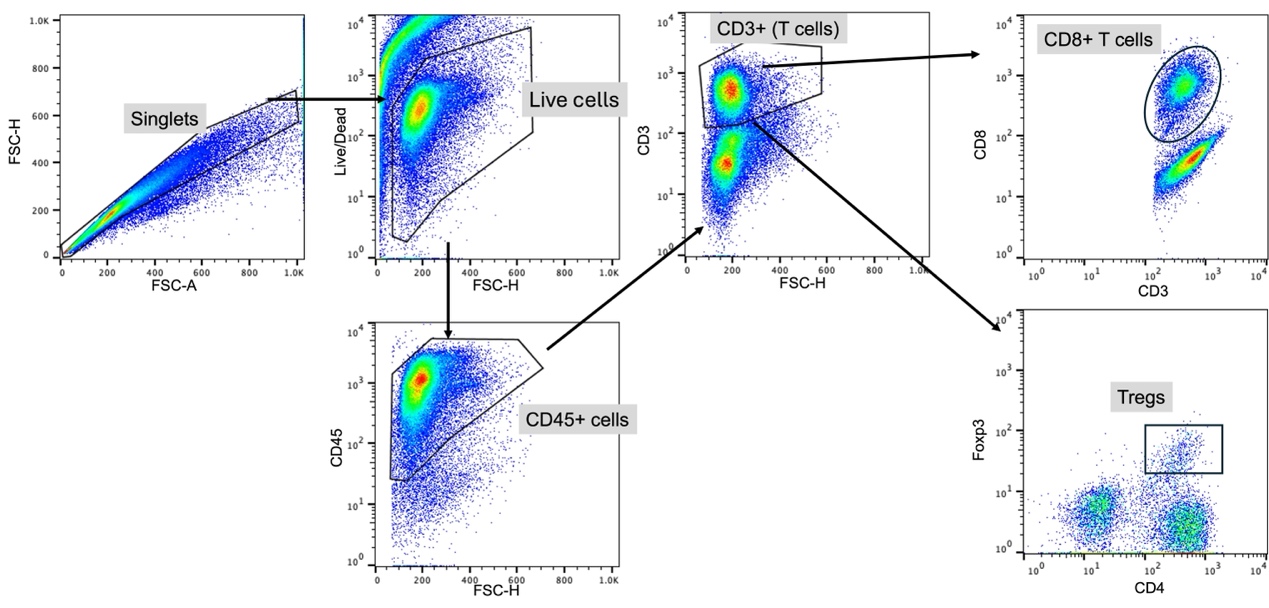


**Figure S40.** Gating strategy for the analysis of liver-infiltrating lymphocytes.


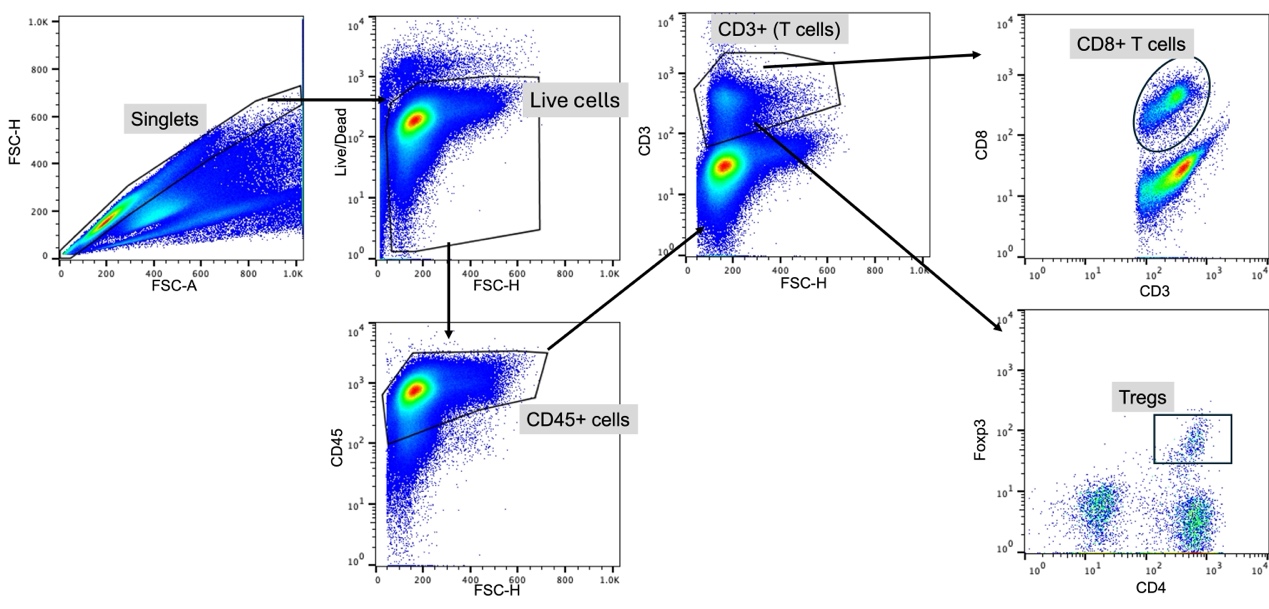


**Figure S41.** Gating strategy for the analysis of blood cells.

**3. References**

[1] Chen, P.; Yang, W.; Nagaoka, K.; Huang, G. Lo; Miyazaki, T.; Hong, T.; Li, S.; Igarashi, K.; Takeda, K.; Kakimi, K.; Kataoka, K.; Cabral, H. An IL-12-Based Nanocytokine Safely Potentiates Anticancer Immunity through Spatiotemporal Control of Inflammation to Eradicate Advanced Cold Tumors. *Advanced Science* **2023**, *10* (10), 2205139.

[2] Chen, P.; Li, S.; Xu, Z.; Cabral, H. Nanoassemblies of heptamethine cyanine dye-initiated poly(amino acid) enhance ROS generation for effective antitumour phototherapy. *Nanoscale Horizons*, **2024**, *9*, 731-741.

[3] Lee, C.; Yang, W.; Parr, R. G. Development of the Colle-Salvetti Correlation-Energy Formula into a Functional of the Electron Density. *Physical Review B: Condensed Matter and Materials Physics* **1988**, *37* (2), 785–789.

[4] J, T.; B, M.; E, C. The IEF Version of the PCM Solvation Method: An Overview of a New Method Addressed to Study Molecular Solutes at the QM Ab Initio Level. *Journal of Molecular Structure* **1999**, *464* (1/3), 211–226.

[5] Hess, B.; Bekker, H.; Berendsen, H. J. C.; Fraaije, J. G. E. M. LINCS: A Linear Constraint Solver for Molecular Simulations. *Journal of Computational Chemistry* **1997**, *18*, 14631472.

[6] Iwai, M.; Pack, C. G.; Takenaka, Y.; Sako, Y.; Nakano, A. Photosystem II Antenna Phosphorylation-Dependent Protein Diffusion Determined by Fluorescence Correlation Spectroscopy. *Scientific Reports* **2013**, *3* (1), 1–7.

[7] Shi, W.; Wang, Y.; Zhang, C.; Jin, H.; Zeng, Z.; Wei, L.; Tian, Y.; Zhang, D.; Sun, G. Isolation and Purification of Immune Cells from the Liver. *International Immunopharmacology* **2020**, *85*, 106632.

[8] J. Derouard, I. Wang, A. Delon, C.E. Leroux, Adaptive optics for fluorescence correlation spectroscopy. *Optics Express*, **2011**, *19*, 26839–26849.
